# The *Penicillium chrysogenum* Q176 Antimicrobial Protein PAFC Effectively Inhibits the Growth of the Opportunistic Human Pathogen *Candida albicans*

**DOI:** 10.3390/jof6030141

**Published:** 2020-08-19

**Authors:** Jeanett Holzknecht, Alexander Kühbacher, Csaba Papp, Attila Farkas, Györgyi Váradi, Jose F. Marcos, Paloma Manzanares, Gábor K. Tóth, László Galgóczy, Florentine Marx

**Affiliations:** 1 Biocenter, Institute of Molecular Biology, Medical University of Innsbruck, Innrain 80–82, A-6020 Innsbruck, Austria; jeanett.holzknecht@i-med.ac.at (J.H.); alexander.kuehbacher@i-med.ac.at (A.K.); 2 Department of Microbiology, Faculty of Science and Informatics, University of Szeged, Közép fasor 52, H-6726 Szeged, Hungary; papp.cs66@gmail.com; 3 Institute of Plant Biology, Biological Research Centre, Temesvári krt. 62, H-6726 Szeged, Hungary; farkas.attila@brc.hu; 4 Department of Medical Chemistry, Faculty of Medicine, University of Szeged, Dóm tér 8, H-6720 Szeged, Hungary; varadi.gyorgyi@med.u-szeged.hu (G.V.); toth.gabor@med.u-szeged.hu (G.K.T.); 5 Department of Food Biotechnology, Instituto de Agroquímica y Tecnología de Alimentos (IATA-CSIC), Consejo Superior de Investigaciones Científicas (CSIC), Paterna, E-46980 Valencia, Spain; jmarcos@iata.csic.es (J.F.M.); pmanz@iata.csic.es (P.M.); 6 MTA-SZTE Biomimetic Systems Research Group, University of Szeged, Dóm tér 8, H-6726 Szeged, Hungary; 7 Department of Biotechnology, Faculty of Science and Informatics, University of Szeged, Közép fasor 52, H-6726 Szeged, Hungary

**Keywords:** *Penicillium chrysogenum* antimicrobial protein C, PAFC, exudate, *Candida albicans*, reactive oxygen species, plasma membrane permeabilization, cell death

## Abstract

Small, cysteine-rich and cationic antimicrobial proteins (AMPs) from filamentous ascomycetes promise treatment alternatives to licensed antifungal drugs. In this study, we characterized the *Penicillium chrysogenum* Q176 antifungal protein C (PAFC), which is phylogenetically distinct to the other two *Penicillium* antifungal proteins, PAF and PAFB, that are expressed by this biotechnologically important ascomycete. PAFC is secreted into the culture broth and is co-expressed with PAF and PAFB in the exudates of surface cultures. This observation is in line with the suggested role of AMPs in the adaptive response of the host to endogenous and/or environmental stimuli. The *in silico* structural model predicted five β-strands stabilized by four intramolecular disulfide bonds in PAFC. The functional characterization of recombinant PAFC provided evidence for a promising new molecule in anti-*Candida* therapy. The thermotolerant PAFC killed planktonic cells and reduced the metabolic activity of sessile cells in pre-established biofilms of two *Candida* *albicans* strains, one of which was a fluconazole-resistant clinical isolate showing higher PAFC sensitivity than the fluconazole-sensitive strain. Candidacidal activity was linked to severe cell morphology changes, PAFC internalization, induction of intracellular reactive oxygen species and plasma membrane disintegration. The lack of hemolytic activity further corroborates the potential applicability of PAFC in clinical therapy.

## 1. Introduction

The global incidence of fungal infections and the increasing resistance development pressure the medical community to find alternatives to the currently used antimycotics. It is estimated that over 300 million patients are affected by fungal infections and around 1.6 million deaths are caused by fungal diseases annually [1].

The most prevalent human pathogenic yeasts which can cause life-threatening infections are the *Candida* species, *Candida albicans* being the most prevalent one, but the incidence of infections caused by non-*albicans Candida* species (NAC) is also increasing [2,3]. *C. albicans* can cause a wide range of human infections, from recurring superficial nail, skin and mucosal infections to severe and life-threatening systemic mycoses [4].

A limited number of licensed antifungal agents are available primarily due to the low number of drug targets that are unique in fungi. This poses a challenge in the prevention and treatment of fungal infections. Prolonged exposure to the currently available antifungal drugs can cause severe side-effects and permanently damage the patients organs [5]. Current anti-*Candida* therapies include azoles, echinocandins or amphotericin B, however the intensive application of azoles in clinical treatment facilitates resistance development in these pathogens [6]. It is of utmost importance to identify new antifungal agents with mechanisms of action that differ from those of existing drugs, with targets that are unique to the fungal cell in order to be safely applicable to humans, and with low risk for resistance development.

A promising source of new anti-*Candida* compounds are antimicrobial proteins (AMPs), secreted by filamentous fungi of the class Eurotiomycetes [7]. One representative of this group, the antibiotic producer *Penicillium chrysogenum*, harbors three genes that code for two well-characterized AMPs—the *P. chrysogenum* antifungal protein (PAF) and antifungal protein B (PAFB) [8]—and a third putative antifungal protein C (PAFC, Pc21g12970) [9]. Amino acid (aa) similarity analysis revealed that PAFC is identical to the Pc-Arctin from an artic *P. chrysogenum* A096 isolate [10] and shares high similarity with the “bubble protein” (BP) from *Penicillium brevicompactum* [11] and the *Penicillium expansum* antifungal protein PeAfpC [12]. PAFC, Pc-Arctin, BP and PeAfpC belong to the so-called “BP cluster”, which is named according to the first reported protein (BP) of this group. They are phylogenetically different to the members of the PAF cluster that includes the two related *P. chrysogenum* AMPs, PAF and PAFB [11,13]. The members of the BP cluster, however, are still less studied in terms of antifungal activity and potential for application compared to other AMPs from filamentous ascomycetes.

The aim of this study was to characterize the antimicrobial protein PAFC of *P. chrysogenum* strain Q176, a descendant of the original penicillin producer strain NRRL1951 and the ancestor of the Wis54-1255 strain whose genome was sequenced [14,15]. For simplicity, we refer in the following to *P. chrysogenum* Q176 as *P. chrysogenum*. We present an *in silico* analysis of the structural properties of PAFC and, we report for the first time the gene expression of a BP cluster protein in submersed cultures and surface colonies, its recombinant production and purification in high yield, and its growth inhibition efficacy against the opportunistic human pathogen *C. albicans*. We have included in the analysis the fluconazole sensitive *C. albicans* strain CBS 5982—referred in the following as *C. albicans^fluS^*—and the fluconazole resistant clinical isolate 27700 (*C. albicans^fluR^*) [16]. We report that both strains were susceptible to PAFC, with *C. albicans^fluR^* being more sensitive than *C. albicans^fluS^*. We have determined the antifungal efficacy of PAFC against planktonic and sessile cells, the cellular uptake of PAFC, the induction of intracellular reactive oxygen species (iROS) and the permeabilization of the plasma membrane to the impermeable cell death indicating dye propidium iodide (PI). The anti-*Candida* efficacy, thermotolerance and absence of hemolytic activity suggest that PAFC may be a potential candidate next generation antifungal drug that is also effective against azole-resistant *C. albicans* strains.

## 2. Materials and Methods

### 2.1. In Silico Structural Prediction of PAFC

*In silico* structural prediction was done based on the hypothetical protein annotated as Pc21g12970 (National Center for Biotechnology Information (NCBI), accession number XP_002568323) in *Penicillium rubens* Wisconsin 54-1255, which is a descendent of *P. chrysogenum* Q176 [14,15]. Full-length protein aa sequence was submitted to the I-Tasser server [17] for prediction of the secondary and tertiary protein structure. The crystal structure of the *P. brevicompactum* BP (PDB ID: 1uoy.pdb) was used as template without alignment, due to high primary structure similarity. The resulting model was chosen based on the Z-score and quality was energy minimized and refined with the ModRefiner [17]. The reliability of the calculated model was evaluated in Ramachandran plot analysis using a RAMPAGE server [18]. The disulfide bond pattern was predicted with Disulfind [19]. To evaluate the reliability of the predicted disulfide bond pattern, the distance between the bonded cysteine residues was analyzed by X-walk [20] using the BP bond pattern as template. Protein model visualization was performed with UCSF ChimeraX software [21]. Alignment of PAFC, PeAfpC and BP was realized with Jalview 2 [22] and sequence colored according to ClustalX. The percent identity matrix between these three proteins was calculated with ClustalW2 Multiple Sequence Alignment tool [23]. The physicochemical properties of PAFC were determined with GRAVY calculator (available online: www.gravy-calculator.de), ProtParam tool (available online: www.web.expasy.org), and ProteinCalculator v3.4 (available online: www.protcalc.sourceforge.net).

### 2.2. Strains and Growth Conditions

Fungal strains used in this study are listed in Supplementary Materials, Table S1 and the composition of the media is described in Supplementary Materials, Table S2. For the generation of conidia, *P. chrysogenum* Q176 was grown on 1 × *Pc*MM for 96 h at 25°C. Conidia were harvested and washed in spore buffer (0.9% NaCl (*w*/*v*), 0.01% Tween (*v*/*v*)) before use. Germlings were generated by growing conidia in 1 × *Pc*MM at 25°C in static culture until the germ tubes were twice the length of the conidia diameter (11 h). For *P. chrysogenum* submersed shaking cultures 200 mL 1 × *Pc*MM was inoculated with 2 × 10^8^ spores and cultivated for up to 96 h at 25 °C and 200 rpm. Synchronized surface cultures were generated on 1 × *Pc*MM, containing 1.5% (*w*/*v*) agar at 25 °C as described in Hegedüs et al., 2011 [24]. For the generation of fungal exudates, 5 μL aliquots containing 1 × 10^6^ *P. chrysogenum* conidia were point inoculated on 1 × *Pc*MM or double-concentrated *Pc*MM (2 × *Pc*MM), containing 1.5% (*w*/*v*) agar. The surface cultures were grown for up to 144 h at 25 °C in a box lined with wet paper towels. Single colonies of *Candida* strains grown on potato dextrose broth (PDB) agar plates (Sigma-Aldrich, St. Louis, MO, USA) were used to inoculate 10 mL ten-fold diluted PDB (0.1 × PDB). After overnight cultivation at 30 °C and 160 rpm the cells were washed in 0.1 × PDB before experimental use. 

### 2.3. Detection of PafC mRNA

The expression of *pafC* was investigated in conidia, germlings, and mycelia of submersed and synchronized surface cultures of *P. chrysogenum*. Total RNA was extracted from cells with TRI Reagent (Sigma-Aldrich, St. Louis, MO, USA) according to the manufacturer’s instruction. Ten µg RNA per lane were loaded onto a 1.2% (*v*/*v*) formaldehyde gel, electrophoresed and blotted onto a Hybond N membrane (Amersham Biosciences, Little Chalfont, UK). The *pafC* transcripts were detected with a digoxigenin-labelled hybridization probe (Roche, Basel, Switzerland) amplified by PCR (Table S3) from *pafC* cDNA using *pafC* specific primers (Table S4).

### 2.4. Detection of Native PAFC

Submersed cultures of *P. chrysogenum* were prepared as described above and the supernatant was used for Western blot analysis at different time points. Fungal exudates, the liquid secreted underneath the fungal colony and the droplets on top of the colony, were harvested as described in Supplementary Materials (experimental procedures; Figure S1) after 120 h of incubation. Samples (20 µL per lane) were loaded onto a 18% (*w*/*v*) SDS-polyacrylamide gel, electrophoresed and Western blotting performed as described (Supplementary Materials, experimental procedures). PAFC was detected using rabbit anti-PeAfpC serum (1:2500) [12]. PAF and PAFB in the exudate were detected using IgG purified rabbit anti-PAF serum (1:500) [25] and rabbit anti-PAFB serum (1:1000) [9], respectively (Supplementary Materials, experimental procedures).

### 2.5. Generation of Recombinant PAFC

Recombinant PAFC was prepared using a *P. chrysogenum*-based expression system [26]. The cloning of expression vector pSK275_*pafC* (Figure S2) and transformation into the *P. chrysogenum* ∆*paf* mutant strain [26] are described in Supplementary Materials (experimental procedures). The strain with the highest PAFC content in the culture broth was named *P. chrysogenum^OEpafC^* and used for large-scale expression. The cell-free supernatant was processed as described previously [26] with minor modification. The pH of the supernatant was adjusted to 4.0 with 1 M citric acid before it was filter sterilized, degassed and loaded onto a BioPro S30 cation exchange column (YMC CO. LTD, Kyoto, Japan). The column was washed with 0.1 M equilibration buffer (0.1 M citrate buffer pH 4, 0.5 M EDTA, 25 mM NaCl) and PAFC was eluted with 0.1 M elution buffer (0.1 M citrate buffer pH 4, 100 mM NaCl). Fractions containing the protein peak were checked for PAFC content and purity on a 18% (*w*/*v*) SDS-polyacrylamide gel and by silver staining. The pure PAFC containing fractions were concentrated and dialyzed against ddH_2_O using a Vivaspin® 500 device (3 kDa MWCO; GE Healthcare, Chicago, IL, USA). The identity of PAFC was verified by electrospray ionization mass spectroscopy (ESI-MS) (Protein Micro-Analysis Facility; Biocenter, Medical University of Innsbruck) and the purity of the protein confirmed by reverse-phase high-performance liquid chromatography (RP-HPLC). The RP-HPLC run was performed on a Phenomenex Jupiter 10 µM C18 300 Å column using the following eluent system: (A) 0.1% (*v*/*v*) trifluoroacetic acid (TFA); (B) 80% (*v*/*v*) acetonitrile and 0.1% (*v*/*v*) TFA. A linear gradient of 15–40% (B) in 25 min was applied at a flow rate of 1.0 mL·min^−1^.

### 2.6. Determination of Anti-Candida Activity

#### 2.6.1. Broth Microdilution Assays

For determination of the inhibitory concentration that reduces growth ≥90% (IC_90_), 100 μL of yeast cells (1 × 10^4^ mL^−1^) were mixed with 100 μL of PAFC in increasing concentrations (0–20 µM) in 0.1 × PDB in 96-well, flat-bottom microtiter plates (Thermo Scientific, Waltham, MA, USA). Amphotericin B, nystatin and fluconazole were purchased from Sigma-Aldrich (St. Louis, MO, USA) and tested in a concentration range of 0–125 µg mL^−1^. To study the effect of serum and ions on the antifungal activity, increasing concentrations of heat-inactivated fetal calf serum (5–15%) and NaCl, CaCl_2_ and MgCl_2_ (1.25–10 mM) were included in broth microdilution assays together with 1 × IC_90_ PAFC and *C. albicans* in 0.1 × PDB as described above. *C. albicans* cells exposed to the supplements without PAFC served as controls. The thermal tolerance of PAFC was studied by incubating the protein for 5 min at 95 °C and determining the antifungal activity immediately after cooling to 25 °C. PAFC that had not been exposed to serum, ions or heat served as full-activity control. The optical density (OD_620nm_) was determined after static cultivation for 24 h at 30 °C using a multi-mode microplate reader (FLUOstar Omega, BMG Labtech, Ortenberg, Germany) operating in well-scanning mode. All assays included a blank (medium without cells) and an untreated growth control representing 100% growth. All experiments were done using technical triplicates and repeated at least twice.

#### 2.6.2. Determination of Colony Forming Units (CFU)

To determine the killing efficacy of PAFC, *C. albicans* planktonic cells from an overnight culture were diluted to 2 × 10^5^ cells·mL^−1^ in 0.1 × PDB and treated with 1 × IC_90_ (2.5 µM), 2 × IC_90_ (5 µM) or 4 × IC_90_ (10 µM) PAFC for 1 h, 8 h and 24 h at 30 °C under continuous shaking (160 rpm). Untreated cells were used as a growth control. Cells were collected by centrifugation, resuspended in 1 mL 0.1 × PDB and a serial dilution up to 10^−6^ was prepared. From each dilution, 100 µL were streaked onto 2% (*w*/*v*) PDB agar plates and incubated at 30 °C for 24 h before the colony forming units (CFU) were counted. The CFU of the untreated control was set at 100%. All experiments were done using technical quadruplicates and repeated twice.

To evaluate the activity of PAFC on sessile cells of *C. albicans,* biofilm formation was induced by distributing 100 μL aliquots of a cell suspension (1 × 10^6^ cells·mL^−1^ in 0.1 × PDB) in the wells of a 96-well, flat-bottom microtiter plate (Thermo Scientific, Waltham, MA, USA) followed by a static incubation for 24 h at 30 °C. The resulting biofilm was checked microscopically for cell attachment and pseudo-hyphae formation before treatment. The biofilm was gently washed with 0.1 × PDB before 100 µL of 1 × IC_90_ (2.5 µM) and 10 × IC_90_ (25 µM) PAFC was added in respective wells. For the negative control, the cells were incubated with 0.1 × PDB and for the positive control the cells were exposed to 10 µg·mL^−1^ amphotericin B. After 24 h of incubation, the biofilm was disrupted by vigorous pipetting, and 10 µL of the detached cells were mixed with 40 µL fresh 0.1 × PDB. Serial dilution of this sample up to 10^−6^ were made and 100 µL streaked out onto 2% (*w*/*v*) PDB agar plates and incubated at 30 °C for 24 h for CFU determination. The CFU of the untreated control was set at 100%. All experiments were conducted using technical triplicates and repeated twice.

### 2.7. Scanning Electron Microscopy (SEM) 

*C. albicans* cells (5 × 10^5^, diluted in 0.1 × PDB) from an overnight liquid culture were incubated with 1 × IC_90_ PAFC for 1 h, 12 h, and 24 h at 30 °C under continuous shaking at 160 rpm. Untreated cells were used as a negative control. Samples were washed, resuspended in phosphate buffered saline (PBS) and 8 µL samples spotted onto a silicon disc coated with 0.01% (*w*/*v*) poly-l-lysine (Merck Millipore, Billerica, MA, USA). Cells were fixed with 2.5% (*v*/*v*) glutaraldehyde and 0.05 M cacodylate buffer (pH 7.2) in PBS overnight at 4 °C. The discs were washed twice with PBS and dehydrated with a graded ethanol series (30%, 50%, 70%, 80%, 100% ethanol (*v*/*v*), for 1.5 h each at 4 °C). Samples of untreated cells served as negative control. The samples were dried with a critical point dryer, followed by 12 nm gold coating (Quorum Technologies, Laughton, East Sussex, UK) and observed under a JEOL JSM-7100F/LV scanning electron microscope (JEOL Ltd., Tokyo, Japan).

### 2.8. Fluorophore-Labeling of PAFC

For visualization in uptake studies PAFC was labeled with the green fluorophore BODIPY™ FL EDA (Bd) (Invitrogen, Carlsbad, CA, USA) as described previously [27]. In brief, 0.4 mM PAFC was labeled with 10 mM Bd in the presence of 10 mM EDAC and 5 mM Sulfo-NHS (Invitrogen, Carlsbad, CA, USA) in 100 M MES-buffer (pH 4.5). The reaction mixture was incubated with continuous shaking at 200 rpm overnight at 25 °C in the dark. The labeled AMP (PAFC-Bd) was dialyzed against ddH_2_O to remove excess Bd and concentrated using Amicon®Ultra centrifugal filters (3 kDa MWCO; Merck Millipore, Burlington, MA, USA). The antifungal activity of PAFC-Bd was tested by broth microdilution assay as described above.

### 2.9. Confocal Laser Scanning Microscopy (CLSM)

*Candida* cells adjusted to 2 × 10^5^ cells·mL^−1^ in 0.1 × PDB were treated with 1 × IC_90_ PAFC-Bd (2.5 µM) for 3 h and 6 h at 30 °C under continuous shaking at 160 rpm. Untreated cells were used as negative control. Samples were washed with PBS and stained consecutively with 5 µg mL^−1^ PI (Sigma-Aldrich, St. Louis, MO, USA) and 5 µg·mL^−1^ calcofluor white (CFW) (Sigma-Aldrich, St. Louis, MO, USA) for 10 min at room temperature in the dark. After washing with PBS, samples were mounted on a glass slide, covered with 2% (*w*/*v*) agar slices and observed with an Olympus Fluoview FV 1000 confocal laser microscope with 60× magnification objective (Olympus, Shinjuku, Japan). A 488 nm laser was used for excitation. The excitation and emission wavelengths were 380 nm and 475 nm for CFW, 504 nm and 512 nm for PAFC-Bd and 535 nm and 617 nm for PI, respectively. Sequential scanning was used to avoid crosstalk between the fluorescent dyes.

### 2.10. Fluorescence Activated Cell Sorting (FACS)

*Candida* cells diluted to 2 × 10^5^ cells·mL^−1^ in 0.1 × PDB were exposed to 1 × IC_90_ PAFC for 1 h, 8 h and 24 h at 30 °C under continuous shaking at 160 rpm. Untreated cells were used as negative control and 70% (*v*/*v*) EtOH treated cells as positive control for PI staining. The cells were washed in PBS, collected by centrifugation at 11,000× *g* for 5 min and stained with 5 µg·mL^−1^ PI for 10 min at room temperature. PI positive cells were counted by a FlowSight imaging flow cytometer (Amins, Merck Millipore, Billerica, MA, USA) with at least 1000 cells were counted per run. For data analysis, the Image Data Exploration and Analysis software (IDEAS; Amins, Millipore, Billerica, MA, USA) was applied. Gates for the data analyses were established according to the unstained control. Experiments were performed using independent triplicates.

### 2.11. Hemolytic Activity

The hemolytic potential of PAFC was evaluated on Columbia blood agar plates (VWR, Randnor, PA, USA). Sterile filter discs (Ø6 mm) were put onto agar plates and loaded with 10 µL aliquots containing 13 µg PAFC. Sterile water and 20% (*v*/*v*) Triton X-100 (10 µL each) served as controls. Plates were incubated at 37 °C for 24 h before documentation.

### 2.12. Statistical Analysis

Data analyses were conducted with Microsoft Excel (2016, Version 16.16.16) software (Microsoft, Redmond, WA, USA). For the calculation of significant differences between the data obtained from treated samples *vs*. the untreated controls, a two-sample Student’s *t*-test was applied. *p*-values of ≤0.05 were considered as significant and *p*-values of ≤0.005 were considered as highly significant.

### 2.13. Image Processing

Images were processed and edited with Axiovision (Carl Zeiss GmbH, Oberkochen, Germany), Fiji [28], GNU Image Manipulation Program (GIMP, version 2.8.10) and Microsoft Power Point (Microsoft Corp.).

## 3. Results

### 3.1. In Silico Prediction of the PAFC Structure

**Figure 1:**
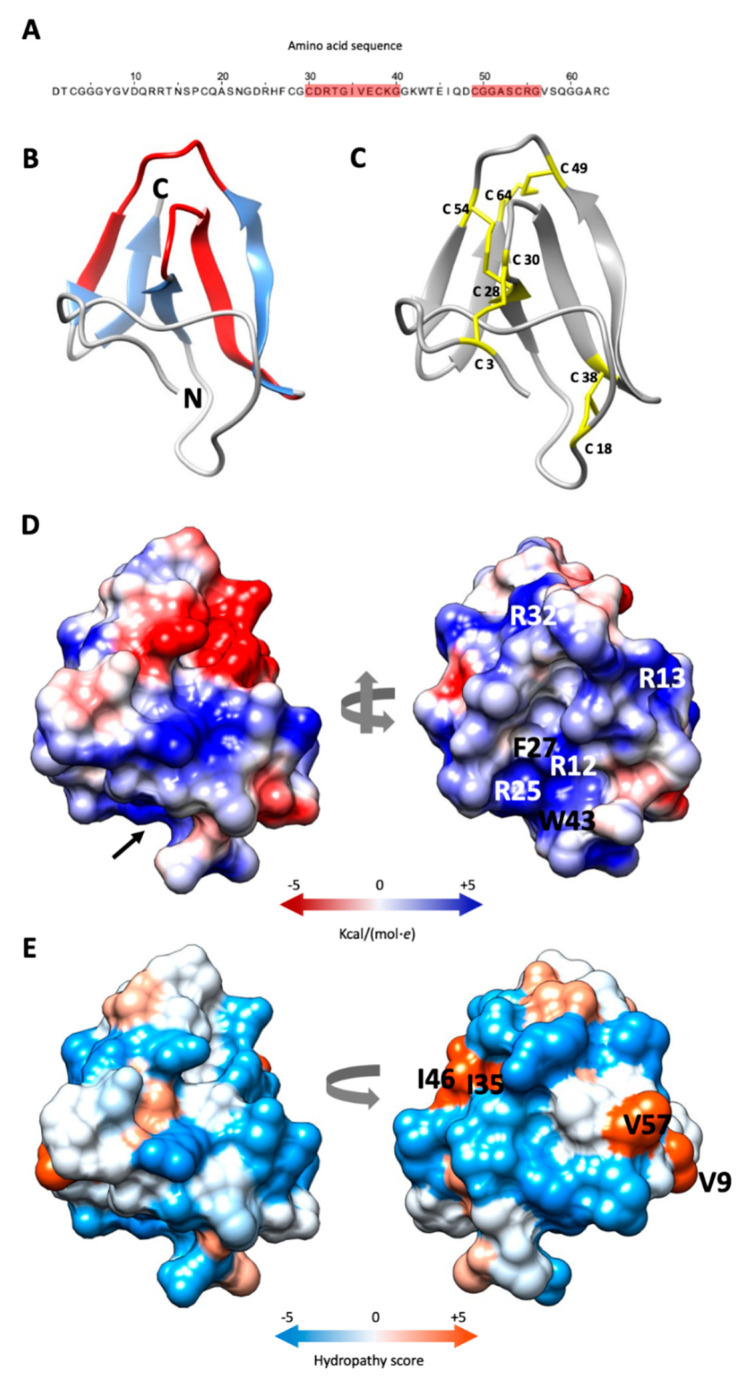
Predicted structure of PAFC. (**A**) Amino acid sequence (aa) of PAFC with the two predicted γ-core motifs highlighted in red. (**B**) Ribbon representation of PAFC in UCSF Chimera protein visualization and analysis software [21] depicting the structure of the β-strands (blue) and location of the two γ-cores (red), the N- and C-terminus is indicated. (**C**) Disulfide bonding of PAFC is shown with yellow sticks. (**D**) Surface representation of PAFC in two orientations colored according to electrostatic potential (blue: electropositive, red: electronegative). The model depicted on the right side visualizes the “mouth-like” cavity indicated by a black arrow in the left model. The basic aas surrounding the opening and the aa forming the funnel base are indicated. (**E**) Distribution of hydrophobic and hydrophilic patches on the surface of PAFC in two orientations colored according to the Kyte-Doolittle scale (blue: hydrophilic, orange: hydrophobic). The aas forming the hydrophobic patches are indicated in the model on the right.

The alignment of the pre-mature PAFC pre-pro protein sequence with that of PeAfpC from *P. expansum* and BP from *P. bevicompactum* revealed 85.2% and 70.1% identity, respectively (Figure S3). The mature PAFC is 64 aa long, has a predicted molecular mass of 6630 Da (assuming all cysteines in reduced form) and is 100% identical to the Pc-Arctin of the arctic isolate *P. chrysogenum* A096 [10]. It has 82.8% and 79.7% identity to the mature form of PeAfpC and BP, respectively (Figure S3). All three proteins possess two putative levomeric γ-core motifs (CX_3-9_CXGX_1-3_) [29], which are conserved among the ascomycetous AMPs of the BP cluster [13]. One of them—positioned in the center of PAFC (CDRTGIVECKG)—is highly conserved, and the second, shorter one with lower homology resides near the C-terminus (CGGASCRG) (Figure 1A and Figure S3).

The *in silico* prediction of the PAFC tertiary structure gave a model showing high fold similarity to the BP of *P. brevicompactum* [30]. The Ramachandran plot of the model showed 95.2% of the aa positioned in energetically favored regions and three in the allowed regions (4.8%) (Figure S4). The PAFC model exhibits five antiparallel β-strands (β1–β5), spanning His26–Cys28 (β1), Gly34–Lys39 (β2), Lys42–Asp48 (β3), Arg55–Val57 (β4) and Gly60–Arg63 (β5), which are connected by four loops (L1–L4). The protein model shows an N-terminal three stranded β-sheet (β-sheet 1) with an N-terminal extended structure and a C-terminal two stranded β-sheet (β-sheet 2). The γ-core motif in the protein center (Cys30–Lys39) encompasses L1 and β2, and the shorter γ-core motif (Cys49–Gly56) contains a part of L3 and β4 (Figure 1B). As already shown for the BP protein [11] the β-pleated structure of PAFC is stabilized by four intramolecular disulfide bonds that are formed between the eight cysteine residues. The predicted disulfide bond pattern (Cys 3/30, Cys 18/38 Cys 28/54, Cys 49/64) of PAFC follows the *abcabdcd* pattern of the BP [30]. The disulfide bonds Cys 3/30 and Cys 18/38 connect the β-sheet 1 to the N-terminal extended region, while Cys 28/54 and Cys 49/64 connect β-sheet 1 with β-sheet 2 (Figure 1C).

Analysis of the electrostatic surface distribution according to Coulomb’s law in UCSF Chimera software [21] indicated that the putative PAFC structure has a cavity similar to BP [30]. The opening of this “mouth-like” structure is dominated by basic aas (Arg12, Arg13, Arg25, Arg32) that are in highly conserved positions in BP and PAFC, with Arg12, Phe27 and Trp43 form the funnel base (Figure 1D). The opposite side has two negatively charged patches, a smaller one consisting of Asp24 and Glu37, and a bigger one, consisting of Asp31, Asp48 and Glu45 (Figure 1D; Supplementary Materials, Video S1). According to the Kyte-Doolittle scale [31], PAFC shows an amphipathic surface with the aas Val9, Ile35/Ile46 and Val57 forming three hydrophobic patches (Figure 1E; Supplementary Materials, Video S2).

### 3.2. Expression of PAFC in P. chrysogenum

**Figure 2:**
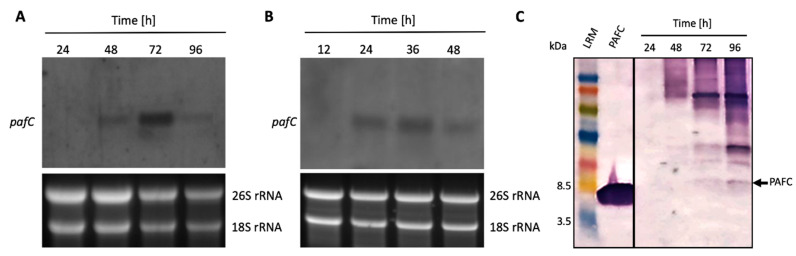
Expression of the PAFC encoding gene and protein. Total RNA was extracted from (**A**) mycelium grown in submersed and (**B**) synchronized surface cultures up to 96 h. Ten μg of total RNA was loaded per lane on a 1.2% denaturing agarose gel, blotted onto a nylon membrane and hybridized with a *pafC* specific digoxigenin-labelled PCR probe (upper panels). Ethidium bromide-stained 26S and 18S rRNA provide loading controls (lower panels). (**C**) For Western blot analysis, 20 μL of the cell free supernatant from submersed cultures was loaded per lane, size fractionated on an 18% (*w*/*v*) SDS-polyacrylamide gel and transferred onto a nitrocellulose membrane. A polyclonal antibody generated against PeAfpC was used to probe for PAFC. Recombinant PAFC (0.5 μg) was used as a control for antibody binding. The black arrow indicates PAFC (6.6 kDa). LRM, low range rainbow marker (GE Healthcare Life Sciences, Little Chalfont, UK). The molecular weight marker bands of 3.5 kDa and 8.5 kDa are indicated.

**Figure 3:**
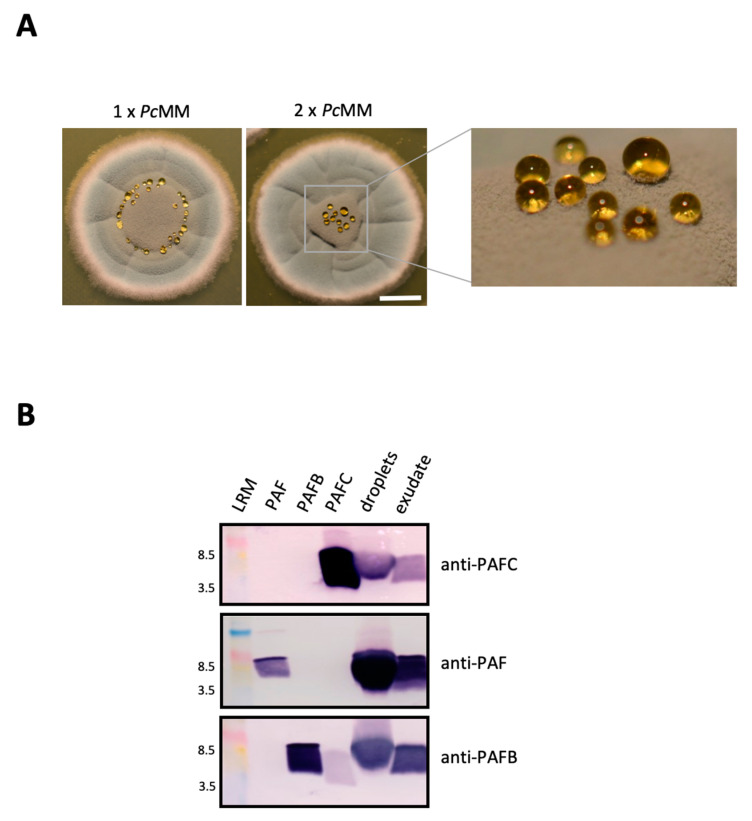
Detection of PAFC in *P. chrysogenum* exudate. (**A**) Droplet formation on *P. chrysogenum* surface cultures grown on 1 × *Pc*MM and 2 × *Pc*MM for 120 h at 25 °C. Scale bar, 5 mm. (**B**) For Western blot analysis, 20 μL of droplets and exudate from cultures grown on 2 × *Pc*MM were loaded per lane, fractionated on an 18% (*w*/*v*) SDS-polyacrylamide gel and transferred onto a nitrocellulose membrane. Polyclonal antibodies were used to detect PAFC, PAF and PAFB. As a control for antibody binding, 0.5 μg of the respective recombinant protein (PAFC, PAF and PAFB) was loaded per lane. LRM, low range rainbow marker (GE Healthcare Life Sciences, Little Chalfont, UK). The molecular weight marker bands of 3.5 kDa and 8.5 kDa are indicated.

Northern blot experiments indicated that the expression of the PAFC encoding gene (*pafC*) peaked after 72 h of cultivation under standard submersed conditions (Figure 2A). Expression of *pafC* was also detected in synchronized *P. chrysogenum* surface cultures and correlated with the onset of conidiation (Figure S5), with gene expression reaching its maximum after 36 h of incubation (Figure 2B). No *pafC* expression was detected in conidia or 11-h-old germlings. These results imply that PAFC is produced under submersed and surface growth conditions in *P. chrysogenum*. To demonstrate PAFC in the fungal culture broth, we performed a Western blot analysis (Figure 2C) probed with a rabbit polyclonal antibody generated against the PAFC-related PeAfpC [12]. This antibody recognized the purified recombinant PAFC (Figure S6) and did not bind to the purified recombinant PAF or PAFB (Figure 3B, upper panel). A faint band with the molecular weight corresponding to mature native PAFC was detected in the crude fermentation broth of submersed cultures after 72 h and 96 h of cultivation (Figure 2C). However, the anti-PeAfpC antibody also bound to secreted proteins with molecular weight > 6.6 kDa in the crude supernatant. This may be due to (Ι) the polyclonal character of the anti-PeAfpC antibody [12] and (ΙΙ) the low PAFC amount in the culture broth, which necessitated a long development time to obtain a detectable signal intensity for the PAFC specific band.

### 3.3. Expression of PAFC, PAF and PAFB in Fungal Exudates

Seibold et al. (2011) [11] found the BP in exudates of *P. brevicompactum* surface cultures. This prompted us to check the droplets formed on the colony surface of *P. chrysogenum* grown on solid medium. These droplets emerged after 120 h of cultivation on colonies that had been point inoculated on solid *P. chrysogenum* minimal medium (1 × *Pc*MM). On this medium the droplets formed a ring around the center of the *P. chrysogenum* colony (Figure 3A). These droplets originated from the fungal exudate that the colony secreted basally. The amount of liquid underneath the colony and the size of droplets could be augmented by cultivating *P. chrysogenum* on agar containing double-concentrated nutrients (2 × *Pc*MM) (Figure 3A). Here the droplets accumulated on top of the colony. When the exudate that had formed underneath the colony after 96 h was harvested, no further droplets emerged on top of the colony with further incubation (Figure S1), indicating the same origin of the exudate and the droplets. After an extended incubation of 144 h the exudate had completely disappeared (Figure S1). PAFC was detected by Western blot analysis in both, the exudate formed underneath the colony and the droplets (Figure 3B). We also tested the samples for the presence of the other two *P. chrysogenum* AMPs and could detect PAF and PAFB by using polyclonal anti-PAF and anti-PAFB antibodies [9,25] (Figure 3B).

### 3.4. Bulk Production and Purification of Recombinant PAFC

Figure 2C indicates that the amount of native PAFC in the supernatant of *P. chrysogenum* was insufficient for purification and further functional investigations. Therefore, we expressed recombinant PAFC using a *P. chrysogenum*-based expression system [26] by cloning *pafC* into the pSK275 vector between the strong *paf* promoter and the *paf* terminator sequence (Figure S2). The *P. chrysogenum* ∆*paf* strain transformed with linearized pSK275_*pafC* served as cell factory for PAFC expression [32,33]. The strain secreting the highest PAFC quantity (*P. chrysogenum^OEpafC^*) was selected for protein production and PAFC was purified from 96 h cell culture supernatant by one-step cation-exchange chromatography (Figure S7A). A protein yield of up to 105 ± 15 mg L^−1^ fermentation broth was obtained. RP-HPLC gave a single elution peak suggesting high purity of the sample (Figure S7B). The identity of PAFC was verified by ESI-MS. The detected mass of 6622 Da matched the predicted mass of oxidised PAFC which contains four disulfide bonds and lacks the pre-pro sequence (Figure S7C).

### 3.5. Anti-Candida Activity of PAFC in Broth Microdilution Assays

The recombinant PAFC was tested for anti-*Candida* activity in broth microdilution assays and the inhibitory concentration that reduces growth ≥90% (IC_90_) was determined (Table 1). PAFC inhibited the growth of the *Candida* spp. tested (Table S1) at low µM concentrations, and the fluconazole sensitive *C. albicans^fluS^* and a fluconazole resistant clinical isolate *C. albicans^fluR^* exhibited the same PAFC sensitivity (IC_90_ 2.5 µM) (Table 1 and Table S1). For comparison, we also tested the susceptibility of *Candida* spp. for the commonly used antifungal drugs fluconazole, amphotericin B and nystatin (Table 1). The *C. albicans^fluR^* strain still proliferated in the presence of the highest fluconazole concentration tested (125 µg mL^−1^) which confirmed its resistance against this common drug under the experimental conditions applied in this study.

### 3.6. Impact of PAFC on C. albicans Biofilm

We investigated the antifungal effect of PAFC on sessile cells of the most prevalent pathogen in this group using *C. albicans^fluS^* and *C. albicans^fluR^*. A 24-h-old biofilm was treated with 1 × IC_90_ (2.5 µM) and 10 × IC_90_ (25 µM) PAFC because biofilms are generally less accessible to antifungal compounds [34] and the established biofilm contains a higher cell number than the experiments performed with planktonic cells.

The survival of the sessile cells evaluated by CFU determination using a plating assay revealed that PAFC inhibited biofilm formation in a concentration dependent manner. Table 2 shows that the number of viable *C. albicans^fluS^* cells decreased significantly after the 24-h treatment with 10 × IC_90_ PAFC compared to the untreated control. The *C. albicans^fluR^* seemed to be more sensitive because a lower PAFC concentration (1 × IC_90_) significantly increased cell death. This was further aggravated when PAFC was applied at 10 × IC_90_. In both strains, however, a PAFC concentration as high as 10 × IC_90_ could not completely impede biofilm growth as shown with 10 µg mL^−1^ amphotericin B (no growth).

### 3.7. Effect of PAFC on the C. albicans Cell Morphology

**Figure 4:**
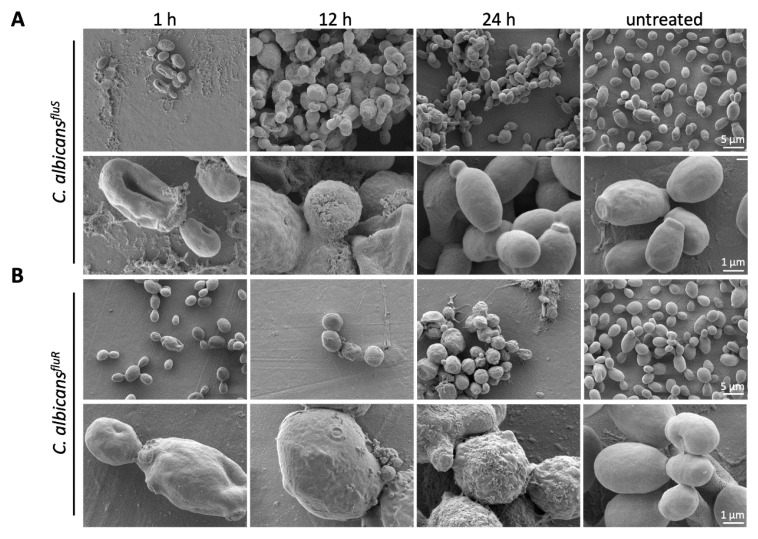
SEM analysis of the impact of PAFC on the *C. albicans* cell morphology. (**A**) *C. albicans^fluS^* and (**B**) *C. albicans^fluR^* cells were incubated with 1 × IC_90_ (2.5 μM) PAFC or without PAFC (untreated) for 1 h, 12 h and 24 h at 30 °C with continuous shaking at 160 rpm. One representative image out of three replicates is shown in overview and high magnification, respectively. Scale bars are indicated in the images.

The effect of PAFC on the morphology of *C. albicans^fluS^* and *C. albicans^fluR^* cells was evaluated using SEM. Untreated control cells exhibited a typical ovoid shape with budding sites and a smooth cell surface (Figure 4). In contrast, a 1 h treatment with 1 × IC_90_ PAFC (2.5 µM) showed cells with a wrinkly surface and amorphous material on and between cells, which may indicate loss of osmotic pressure and leakage of cell contents, respectively. With longer incubation time (12 h), *C. albicans* cells showed severe signs of cell leakage and lost their ovoid shape. After 24 h morphologically intact *C. albicans^fluS^* cells were observed again, suggesting that some cells survived the treatment and resumed growth (Figure 4A). In contrast, *C. albicans^fluR^* cells still appeared severely damaged after 24 h of PAFC exposure (Figure 4B).

### 3.8. Cellular Localization of PAFC and Cell Death Induction

**Figure 5:**
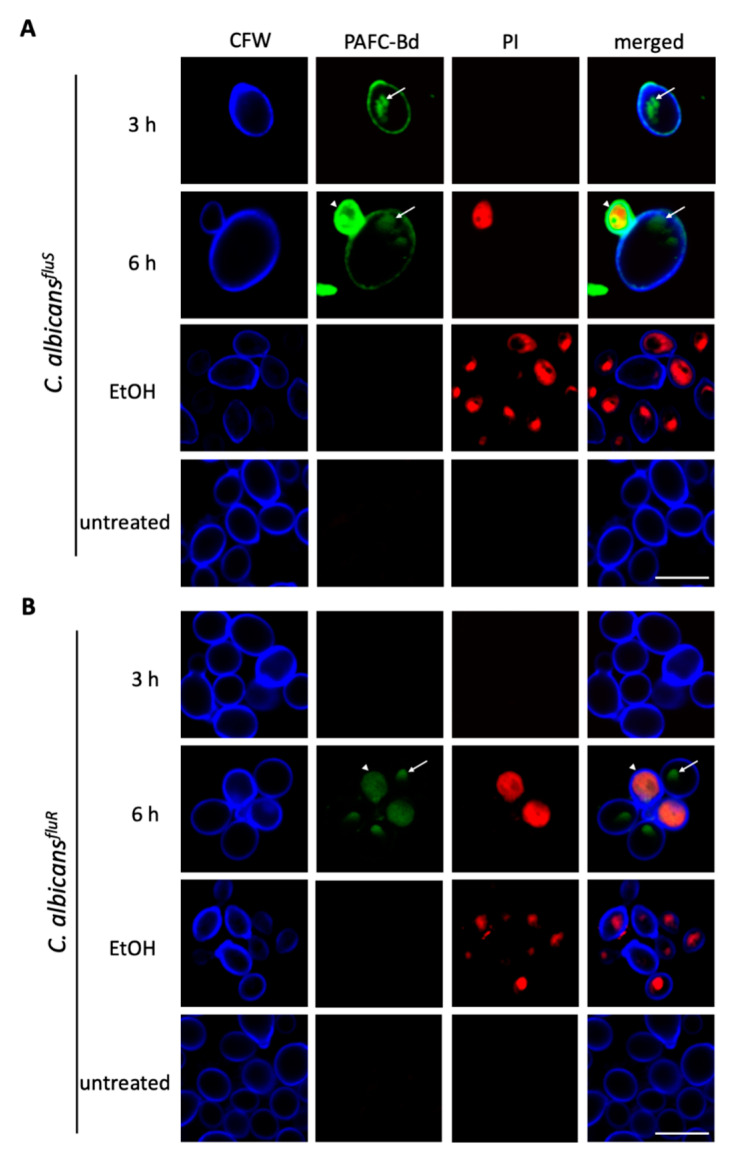
CLSM analysis of the cellular localization of PAFC and cell death induction. Cells of (**A**) *C. albicans^fluS^* and (**B**) *C. albicans^fluR^* were exposed to 1 × IC_90_ PAFC-Bd (2.5 μM) for 3 h, 6 h and 12 h and then stained with calcofluor white (CFW, 5 μg mL^−1^) and propidium iodide (PI, 5 μg mL^−1^) for 10 min. Control cells were left untreated. Sequential scanning was done for CFW (blue), PAFC-Bd (green) and PI (red) at 405, 488 and 543 nm, respectively. White arrows indicate vacuoles, white arrowheads dead cells. One representative image out of three replicates is shown. Scale bar, 5 μm.

To investigate whether PAFC interacts only with the outer cell layers (cell wall or plasma membrane) or enters the cytosol of the *C. albicans* cells we used CLSM to detect PAFC labeled with the green fluorophore BODIPY (PAFC-Bd). The fluorophore-labelling of PAFC had no adverse impact on its antifungal activity. The application of 2.5 µM PAFC-Bd in a broth microdilution assay resulted in *C. albicans* growth inhibition of 98.6%, which corresponded to the IC_90_ of PAFC. Co-staining with the membrane impermeant fluorescent cell death dye PI visualized cells that were killed by the interaction with PAFC-Bd. Cells were incubated for 3 h and 6 h to ensure that the PAFC-Bd specific fluorescence signal was intense enough for visualization. After 3 h PAFC-Bd attached to the outer layers of *C. albicans^fluS^* and this signal co-localized with the cell wall specific blue dye CFW (Figure 5A). Intracellular fluorescent patches (subcellular structures) indicated that PAFC-Bd was taken up by the cells without cell death induction (no PI signal). After 6 h the positively stained cells showed the PAFC-Bd signal dispersed in the whole cell (cytoplasm) that coincided with a strong intracellular PI signal, whereas cells exhibiting a PAFC-Bd signal that still localized in subcellular structures remained PI-negative (Figure 5A). A similar uptake and intracellular staining pattern with PAFC-Bd was observed with *C. albicans^fluR^* cells, although interaction of PAFC-Bd with cells could be detected only at 6 h of incubation (Figure 5B). Localization of PAFC-Bd with the nuclei was excluded when co-staining with the nuclei-specific dye Hoechst 33342 and imaged by fluorescence microscopy (Supplementary Materials, experimental procedures; Figure S8). Taken together, these qualitative microscopy-based data revealed that PAFC first enters sensitive cells before the plasma membrane is compromised and cell death is induced. This suggests that an intracellular PAFC target may exist.

### 3.9. Candidacidal Efficacy of PAFC

The time course of cell death caused by PAFC was detected using the cell death specific dye PI with FACS analysis (Table 3). Exposure of *C. albicans^fluS^* to 1 × IC_90_ (2.5 µM) PAFC resulted in a time dependent increase in the proportion of PI positive cells after 1 h and 8 h of incubation. Interestingly, after a 24 h incubation the number of cells with a PI-positive phenotype dropped below 1%, suggesting that surviving cells had resumed growth. The treatment of *C. albicans^fluR^* with PAFC similarly induced an increase in PI-positive cells between 1 h and 8 h of exposure, though in a slightly delayed manner. In strong contrast to the *C. albicans^fluS^*, however, the percentage of *C. albicans^fluR^* cells with a PI-positive phenotype further increased with time and reached more than 70% after 24 h, indicating that a high proportion of cells suffered from plasma membrane permeabilization due to PAFC treatment.

**Figure 6:**
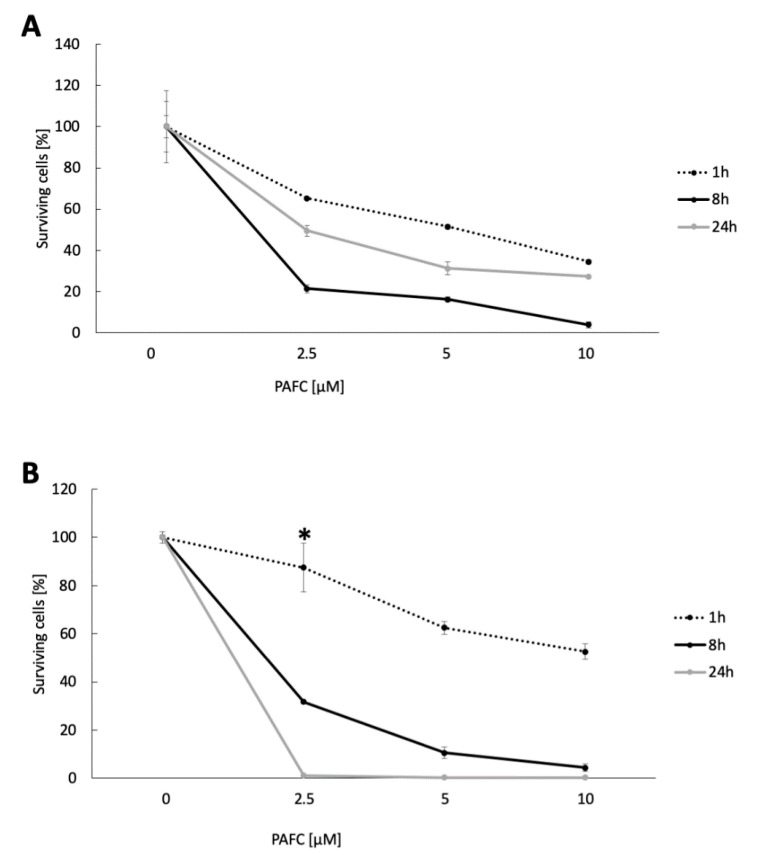
Candidacidal activity of PAFC. CFU of (**A**) *C. albicans^fluS^* and (**B**) *C. albicans^fluR^* cells after PAFC treatment (2.5–10 μM) for 1 h, 8 h and 24 h. Untreated cells served as growth control and the CFU counts were set to be 100% surviving cells. The mean ± SD (technical quadruplicates of one representative experiment out of two biological replicates) is shown. A two-sample Student’s *t*-test was applied to calculate the significant difference between the treated samples compared to the untreated growth control (*p* ≤ 0.005 for all values shown, except for * *p* < 0.1).

These data were confirmed by using CFU determinations after the exposure of *C. albicans* cells to increasing PAFC concentrations (2.5–10 µM) corresponding to 1 × IC_90_, 2 × IC_90_ and 4 × IC_90_ in a time course (1 h, 8 h, 24 h) (Figure 6). In *C. albicans^fluS^*, only 21% ± 2.0% survived the 8-h treatment with 1 × IC_90_, but after 24 h of exposure the CFU increased again to 49.5% ± 2.5%, indicating that surviving cells had resumed growth (Figure 6A). In contrast, 1 × IC_90_ PAFC treatment of *C. albicans^fluR^* gave higher CFU numbers (31.0% ± 0.7%) after 8 h, but very few cells survived the 24-h treatment (1.1% ± 0.001%) (Figure 6B). This time-dependent trend was strain specific and apparent at all concentrations tested. These results comfirm the data generated by FACS and suggest that while PAFC killed *C. albicans^fluS^* cells quickly, the survivors of the treatment proliferated again. In contrast, PAFC had a delayed action on *C. albicans^fluR^* but was ultimately more effective as a fungicide.

### 3.10. Intracellular ROS Induction by PAFC

**Figure 7:**
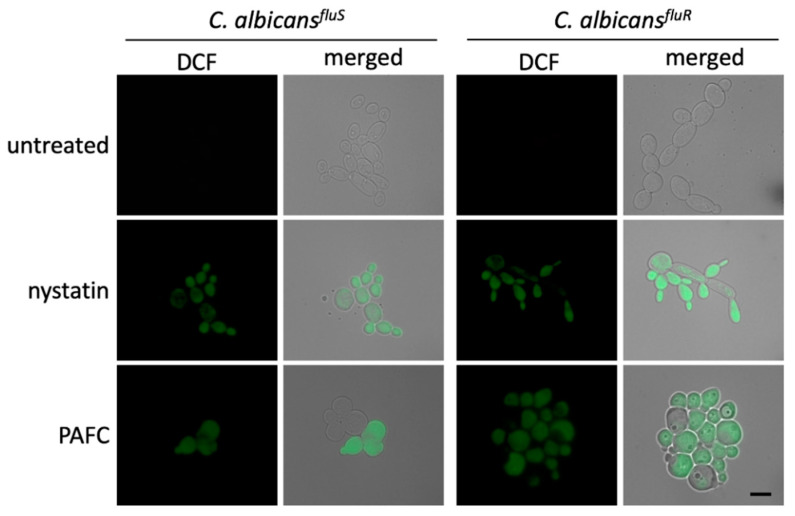
Induction of iROS by PAFC. Fluorescence microscopy of DCF-positive *C. albicans^fluS^* and *C. albicans^fluR^* cells to visualize iROS burden after exposure to 1 × IC_90_ (2.5 µM) PAFC for 8 h at 30 °C and continuous shaking at 160 rpm. The samples were washed in PBS before incubation for 30 min with H_2_DCFDA (5 ng μL^−1^). Excess of fluorescent dye was removed by washing in PBS and the cells were mounted on glass slides for evaluation of iROS induction by fluorescence microscopy. Cells without treatment and exposed to nystatin (10 µg mL^−1^) were used as negative and iROS-positive controls, respectively. The merged images show the DCF signal (green) superimposed with the *Candida* cells visualized with brightfield microscopy. One representative experiment out of three independent experiments is shown. Scale bar, 5 μm.

Studies on the mode of action of fungal AMPs have shown that their activity is often closely linked with the induction of iROS [13,35,36]. The formation of iROS in *Candida* cells in response to PAFC was therefore investigated by fluorescence microscopy (Supplementary Materials, experimental procedures). *C. albicans* cells exposed to 1 × IC_90_ PAFC (2.5 µM) for 8 h were loaded with the non-fluorescent dye dichlorodihydrofluorescein diacetate (H_2_DCFDA). In the presence of iROS, this compound is converted intracellularly to the fluorescent dichlorofluorescein (DCF). As depicted in Figure 7, both *C. albicans* strains suffered from iROS induction by PAFC, just like the positive control cells, which had been exposed to 10 µg mL^−1^ of the ROS inducing polyene drug nystatin. Untreated control cells did not show any DCF specific signal (Figure 7).

### 3.11. Testing of Serum–, Ion–, Thermotolerance and Hemolytic Activity of PAFC

**Figure 8:**
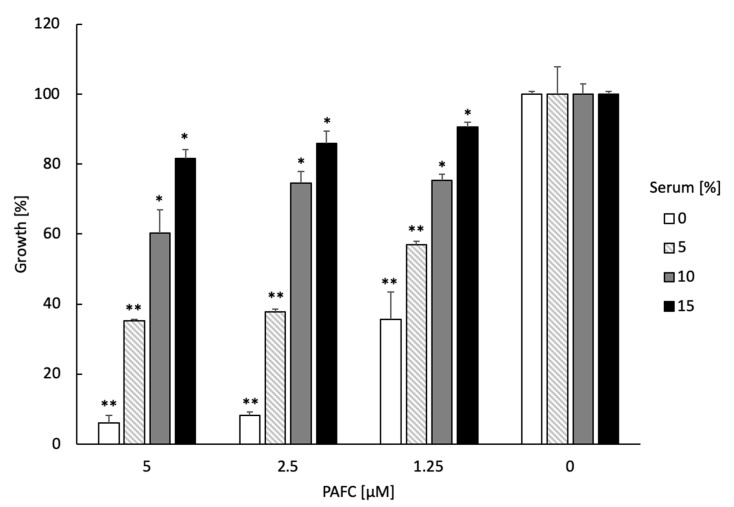
Serum sensitivity of PAFC. The activity of 0.5 × IC_90_ (1.25 µM), 1 × IC_90_ (2.5 µM) and 2 × IC_90_ (5.0 µM) PAFC was tested on *C. albicans^fluS^* in the presence of increasing concentrations (0–15%) of inactivated fetal calf serum in a microdilution broth assay. *Candida* cells without PAFC treatment (0 µM) were used as control representing 100% growth in the presence of 0–15% serum. The mean ± SD (technical triplicate of one representative experiment out of two biological replicates) is shown. A two-sample Student’s *t*-test was applied to calculate the significant difference between the serum-treated samples compared to the untreated growth control (* *p* ≤ 0.05 and ** *p* ≤ 0.005).

The tolerance of PAFC to serum compounds, high ion concentrations and extreme temperature as well as the lack of adverse effects in the host are important prerequisites for considering this AMP for a potential medical application in the future. The presence of 5–15% inactivated fetal calf serum in the medium reduced PAFC activity against *C. albicans^fluS^* in a dose-dependent manner (Figure 8). Similarly, the supplementation of the growth medium with 1.25–10 mM MgCl_2_, NaCl and CaCl_2_ counteracted the PAFC activity in a concentration dependent manner, with PAFC being most sensitive to MgCl_2_ (Figure S9). The heating of PAFC to 95 °C for 5 min and cooling to 25 °C had no adverse effect on its antifungal efficacy with retention of an IC_90_ of 2.5 μM by *C. albicans^fluS^*. PAFC reduced the fungal growth after 24 h of incubation to 7.5 ± 2.7% and to 5.8 ± 4.2% before and after its thermal treatment, respectively, which underlined its high thermotolerance. Finally, PAFC showed no hemolytic activity when tested on sheep erythrocytes in an agar diffusion assay using Columbia blood agar plates (Figure S10).

## 4. Discussion

We have predicted the structure, studied the expression and characterized the antifungal mode of action of PAFC, which phylogenetically belongs to the BP group and represents—apart from PAF and PAFB [8,9,13,37]—the third and previously uncharacterized AMP from the industrial strain *P. chrysogenum* Q176.

Similar to the crystal structure of the *P. brevicompactum* BP, *in silico* analysis of PAFC suggested a β-fold structure containing five antiparallel β-strands. Notably, for PeAfpC, a β-sheet structure composed of only three β-strands was predicted [12]. This discrepancy could result from the different *in silico* approach and prediction software applied to model the proteins. The disulfide bonds in cysteine-rich AMPs mediate high tolerance to harsh environmental conditions [38]. For Pc-Arctin, a high thermal stability but low ion tolerance and low proteolytic stability has been reported [10]. In line with this observation, PAFC showed a high sensitivity to serum and ions but thermal tolerance under the experimental conditions applied. The positively charged funnel-like opening predicted for PAFC could be involved in protein function, such as ligand binding and/or interaction with negatively charged membrane compounds of the fungal target cells, as suggested for this motif in BP [30,39,40]. A detailed analysis by nuclear magnetic resonance to resolve the PAFC solution structure is in progress.

The presence of the *pafC* transcripts in the mycelium of shaking submersed and surface cultures grown on solid medium, but not in dormant conidia or germinated conidia, indicated that PAFC might play a role after colony establishment in fungal growth and/or differentiation of *P. chrysogenum*. This assumption was supported by the finding that *pafC* transcription started in the mycelia of surface cultures at the onset of sporulation.

It is generally hypothesized that AMPs from filamentous fungi—apart from their antifungal activity—fullfill additional functions in the host during adaptation to environmental conditions [41,42]. In *P. chrysogenum* roles of PAF and PAFB were reported in sensing/signaling during growth, differentiation and/or asexual sporulation [8,24,41] but detailed knowledge at the molecular level of the role of fungal AMPs in adaptive responses is still lacking. However, we have shown for the first time that the three *P. chrysogenum* AMPs PAF, PAFB and PAFC are abundantly secreted together into the exudate of old sporulating surface colonies grown on rich medium. Generally, fungal exudates contain secondary metabolites, enzymes and other by-products that are assumed to support cell survival under unfavorable environmental conditions. These liquid reservoirs can be reabsorbed when needed [43]. Indeed, these droplets and the exudate below the colony disappeared with longer incubation. This observation could indicate that exudate production in *P. chrysogenum* is related to nutrient access and the age of the mycelium.

The amount of endogenous PAFC was insufficient to provide the purified protein needed for characterization of its antifungal activity. Instead, we used a well-established *P. chrysogenum*-based expression system to produce recombinant PAFC. We obtained protein yields better than those achieved previously with other recombinant AMPs using this system [9,12,13,26,27,44,45,46,47,48].

The BP cluster AMPs were poorly characterized with regard to their potential medical applicability. Our study shows the *in vitro* growth inhibitory efficacy of a BP cluster protein against opportunistic pathogenic fungi of the genus *Candida*. The anti-*Candida* effective concentration range of PAFC resembled that of PAF and PAFB, the latter showing the highest efficacy against the NAC species *C. glabrata*, *C. krusei* and *C. parapsilosis* (IC_90_ 0.6 μM) with PAF being most effective against *C. parapsilosis* (IC_90_ 2.5 μM) [7,9]. The growth inhibitory activity of Pc-Arctin from *P. chrysogenum* A096 was tested on the common environmental mold *Paecilomyces variotii*, the plant pathogen *Alternaria longipes*, and the biofungicide *Trichoderma viride* [10]. For the *P. brevicompactum* BP, growth inhibitory activity against *Saccharomyces cerevisiae* was reported [11], whereas no antifungal activity was detected for PeAfpC [12].

To dissect in more detail the antifungal mode of action of PAFC, we used two strains of the most prevalent human pathogenic yeast, the fluconazole-sensitive *C. albicans^fluS^* and the fluconazole-resistant clinical isolate *C. albicans^fluR^* [16], whereby the latter strain still proliferated in the presence of the highest fluconazole concentration applied in this study. The molecular basis for the fluconazole resistance of *C. albicans^fluR^* is not known and subject of current investigations.

Both *C. albicans* strains exhibited the same IC_90_ value (2.5 μM). However, the broth microdilution assays show effects on growth but not on the killing potential of an antifungal compound. Therefore, we applied plating assays for CFU determination plus SEM, CLSM and FACS to visualize and quantitate the fungicidal efficacy of PAFC. Our data indicated that PAFC acted in a fungicidal way on sessile and planktonic cells of both *C. albicans* strains under the test conditions applied, though the efficacy was higher against *C. albicans^fluR^* than against *C. albicans^fluS^*. After 24 h of incubation PAFC killed more planktonic and sessile cells of *C. albicans^fluR^* than of *C. albicans^fluS^*. Interestingly, cells of the latter strain resumed growth after 24 h of PAFC exposure, which suggests an adaptive response to PAFC treatment. In contrast, *C. albicans^fluR^* might lack the ability to respond adequately to the antifungal PAFC action [49]. It is known that drug resistance can be acquired at the cost of cellular fitness, leading to a disadvantage for the organism under unfavorable conditions in the absence of the specific drug [50]. Further investigations are needed to clarify, if azole-resistant *C. albicans* strains are generally more susceptible to AMPs than sensitive ones. In this respect, the comparison of several well characterized drug-resistant clinical isolates with standard strains for PAFC susceptibility or the screening of drug-sensitive parental and resistant daughter strains isolated from the same patient could be supportive.

The cell damage induced by PAFC resembled that reported for the candidacidal-acting *Neosartorya fischeri* antifungal protein 2 (NFAP2) [16]. However, our qualitative analysis by CLSM revealed that cell death in *C. albicans* requires PAFC uptake and cytoplasmic localization before plasma membrane permeabilization occurs pointing towards an intracellular target. This mode of action strongly resembles that of *P. chrysogenum* PAF and PAFB [9,25,27], the *N. fischeri* antifungal protein NFAP [48] and synthetic antifungal peptides, e.g., PAF26 [51]. Notably, PAFC activity was closely linked to the accumulation of iROS, which is generally recognized as a trigger for apoptotic cell death in *C. albicans* [52]. Indeed, oxidative stress seems to be a common phenotypic marker of AMP-mediated antifungal activity as reported for PAF [13,53], several plant defensins [54,55] and synthetic antifungal peptides [13,56].

## 5. Conclusions

The emerging drug resistance of human pathogenic fungi and the limited number of antifungal drugs requires the urgent identification of new antifungal compounds with novel mechanism of action [2]. We have shown that PAFC is a promising new antifungal biomolecule. It can be produced in high yields and quality in *P. chrysogenum,* a generally recognized as safe organism [57]. The recombinant PAFC effectively inhibited the growth of the opportunistic human pathogen *C. albicans* and exhibited candidacidal activity with high efficacy against a fluconazole-resistant *C. albicans* clinical isolate. The high thermotolerance and the lack of hemolytic activity further supports its potential applicability in clinical therapy. Similar, to many other cysteine-rich, cationic AMPs from filamentous fungi [8,10,27,53,58] PAFC also exhibited serum and cation sensitivity, which may hamper an intravenous application to combat severe systemic fungal infections. Instead, PAFC might help to develop new drugs for topical prevention or cure of fungal nail, skin and mucosal infections while repeated application of PAFC may be required to maintain its candidacidal efficacy. Furthermore, rational design could be a promising option to improve the antifungal efficacy of PAFC and overcome features that limit its potential for medical application.

## Figures and Tables

**Table 1:** Inhibitory concentrations of PAFC and commonly used antifungal drugs reducing *Candida* spp. growth ≥90% (IC_90_) ^§^.

Microorganisms	PAFC	Fluconazole	Amphotericin B	Nystatin
*Candida albicans^fluS^*	2.50 (16.50)	6.37 (1.95)	0.02 (0.02)	2.11 (1.95)
*Candida albicans^fluR^*	2.50 (16.50)	408.50 (>125)	1.05 (0.97)	0.53 (0.49)
*Candida glabrata*	0.15 (0.99)	12.78 (3.91)	0.03 (0.03)	0.53 (0.49)
*Candida guilliermondii*	3.12 (20.59)	1.60 (0.49)	0.06 (0.06)	1.06 (0.98)
*Candida krusei*	2.50 (16.50)	51.08 (15.63)	0.06 (0.06)	1.06 (0.98)
*Candida parapsilosis*	0.04 (0.26)	12.78 (3.91)	8.45 (7.81)	1.06 (0.98)

^§^*Candida* spp. (1 × 10^3^ cells mL^−1^) were grown in 0.1 × PDB for 24 h at 30 °C under static conditions. IC_90_ was defined as PAFC concentration that inhibits growth ≥90% compared to the untreated control. IC_90_ values are given in µM (µg mL^−1^ in parenthesis).

**Table 2:** Survival of *Candida albicans* sessile cells after 24 h treatment with PAFC determined by plating assay ^§^.

Strain	Untreated	PAFC		Amphotericin B 10 µg mL^−1^
		1 × IC_90_	10 × IC_90_	
*Candida albicans^fluS^*	100 ± 10.8	83.4 ± 3.0	47.0 ± 3.8 *	0 ± 0 **
*Candida albicans^fluR^*	100 ± 5.8	48.1 ± 2.2 **	28.1 ± 4.5 **	0 ± 0 **

^§^ Values of surviving cells are given in % CFU of the untreated control. * *p* ≤ 0.05 and ** *p* ≤ 0.005.

**Table 3:** PI-positive *C. albicans* cells at 1 h, 8 h and 24 h of PAFC treatment evaluated by FACS analysis ^§^.

Strain	Untreated	1 h	8 h	24 h	EtOH
*C. albicans^fluS^*	0.10 ± 0.1	38.10 ± 0.8 **	60.95 ± 12.0 **	0.89 ± 0.3	99.17 ± 3.1 **
*C. albicans^fluR^*	0.09 ± 0.04	17.37 ± 2.3 **	53.30 ± 5.6 **	77.95 ± 11.7 **	99.17 ± 5.4 **

^§^ Values of cells with PI-positive phenotype are given in % of all cells counted (100%). ** *p* ≤ 0.005.

## Supplementary Materials

The following are available online at https://www.mdpi.com/2309-608X/6/3/141/s1, Table S1. Fungal and bacterial strains used in this study; Table S2. Composition of media and solutions used in this study; Table S3. PCR conditions applied in this study; Table S4. Oligonucleotides used in this study; Figure S1. Exudate formation of *P. chrysogenum* surface colonies on 2 × *Pc*MM agar; Figure S2. Cloning of the expression vector pSK275_*pafC*; Figure S3. Amino acid alignment of PAFC and orthologous AMPs; Figure S4. Ramachandran plot of the PAFC model; Figure S5. Expression of *pafC, paf* and *pafB* in synchronized surface cultures of *P. chrysogenum* over a time course of 12–48 h of incubation; Figure S6. Western blot analysis to prove the binding of the PeAfpC antibody to PAFC; Figure S7. Purification of recombinant PAFC; Figure S8. Fluorescence microscopy for the localization of nuclei and PAFC-Bd; Figure S9. Ion tolerance of PAFC; Figure S10. Hemolytic activity of PAFC tested with agar diffusion assay; Video S1. Rotation of the PAFC model colored according to the Coulomb scale (blue: electropositive, red: electronegative); Video S2. Rotation of the PAFC model colored according to the Kyte-Doolittle scale (blue: hydrophilic, orange: hydrophobic).
