# Supplementary material for: The Penicillium chrysogenum Q176 Antimicrobial Protein PAFC Effectively Inhibits the Growth of the Opportunistic Human Pathogen Candida albicans
Source: J Fungi (Basel). 2020 Aug 19;6(3):141. doi: 10.3390/jof6030141 (PMC7557831; doi:10.3390/jof6030141)
Supplement: Supplementary file 1 [file jof-06-00141-s001.zip › jof-879567_Supplements _Revision.pdf]

Supplementary Materials

The *Penicillium chrysogenum* Q176 antimicrobial protein PAFC effectively inhibits the growth of the opportunistic human pathogen *Candida albicans*

Jeanett Holzknecht[1], Alexander Kühbacher[1], Csaba Papp[2], Attila Farkas[3], Györgyi Váradi[4], José F. Marcos[5], Paloma Manzanares[5], Gábor K. Tóth[4,6], László Galgóczy[3,7*] and Florentine Marx[1]**

[1] Biocenter, Institute of Molecular Biology, Medical University of Innsbruck, Innrain 80-82, A-6020 Innsbruck, Austria; jeanett.holzknecht@i-med.ac.at, alexander.kuehbacher@i-med.ac.at, florentine.marx@i-med.ac.at

[2] Department of Microbiology, Faculty of Science and Informatics, University of Szeged, Közép fasor 52, H-6726 Szeged, Hungary; papp.cs66@gmail.com

[3] Institute of Plant Biology, Biological Research Centre, Temesvári krt. 62, H-6726 Szeged, Hungary; farkasa8@brc.com, galgoczi@brc.hu

[4] Department of Medical Chemistry, Faculty of Medicine, University of Szeged, Dóm tér 8, H-6720 Szeged, Hungary; varadi.gyorgyi@med.u-szeged.hu, toth.gabor@med.u-szeged.hu

[5] Department of Food Biotechnology, Instituto de Agroquímica y Tecnología de Alimentos (IATA-CSIC), Consejo Superior de Investigaciones Científicas (CSIC), Paterna, E-46980 Valencia, Spain; pmanz@iata.csic.es, jmarcos@iata.csic.es

[6] MTA-SZTE Biomimetic Systems Research Group, University of Szeged, Dóm tér 8, H-6726 Szeged, Hungary; toth.gabor@med.u-szeged.hu

[7] Department of Biotechnology, Faculty of Science and Informatics, University of Szeged, Közép fasor 52, H-6726 Szeged, Hungary; galgoczi@bio.u-szeged.hu

Correspondence: *galgoczi.laszlo@brc.hu; Tel. +36 62 599 600 (415) (L.G.); **florentine.marx@i-med.ac.at; Tel. +43 512 9003 (70207) (F.M.)

Supplementary Experimental Procedures

Harvest of the fungal exudate

P. chrysogenum colonies grown on 2×*Pc*MM agar for 120 h at 25°C secreted exudate at the bottom of the colony after 96 h and then droplets appeared on top of the colony. The liquid underneath the colony was harvested with a sterile syringe by puncturing the colony and the droplets were collected with a sterile pipette tip (Figure S1). The samples were analyzed for the presence of PAFC, PAF and PAFB by Western blot.

Proof of PeAfpC antibody binding of PAFC and Western blotting

The detection of PAFC was tested in a Western blot using PeAfpC antibody. To exclude cross-reactivity, the *P. chrysogenum* AMPs PAF and PAFB were included, where indicated. Protein samples (0.5-5 µg per lane) were loaded onto an 18% (w/v) SDS-polyacrylamide gel, electrophoresed and blotted onto a 0.2 µm PVDF nitrocellulose membrane (Bio-Rad Laboratories, Inc., Hercules, CA, USA) using the Trans-Blot Turbo Transfer system (Bio-Rad Laboratories Inc., Hercules, CA, USA). The blot was then blocked with blocking buffer (0.3% (v/v) Tween 80, 3% (w/v) skimmed milk powder in PBS (pH 7.4)) for 2 h at room temperature and incubated with IgG purified rabbit anti-PAF serum (1:500) [1], rabbit anti-PAFB serum (1:1000) [2] or rabbit anti-PeAfpC serum (1:2500) [3] in blocking solution (0.3% (v/v) Tween 80, 1.5% (w/v) skimmed milk powder in PBS (pH 7.4)) for 3 h at room temperature. The blot was washed in washing buffer (0.3% (v/v) Tween 80 in PBS (pH 7.4)) three times and incubated with blocking buffer containing the anti-rabbit IgG alkaline phosphatase secondary antibody (1:10000, Sigma-Aldrich, Vienna, Austria) up to 3 h at room temperature. After washing three times in washing buffer and once in ddH$_2$O for 5 min, the blot was equilibrated with alkaline phosphatase substrate buffer (100 mM Tris-HCl (pH 8.3), 150 mM NaCl, 1 mM MgCl$_2$) for 5 min. The signal was developed by addition of p-nitroblue tetrazolium (0.5% (v/v), Promega, Madison, WO, USA) and 5-bromo-4-chloro-3-indolyl phosphate (0.25% (v/v), Promega, Madison, WO, USA) in alkaline phosphatase substrate buffer until desired intensity of the protein bands was achieved. The reaction was stopped with ddH$_2$O for 5 min at room temperature.

Cloning of the PAFC expression plasmid

A PAFC overexpression strain was generated as described in Sonderegger *et al.* (2016) [4]. Briefly, pSK275_*pafB* [2] was digested with XmaI and BglII to excise the *pafB* gene and replace it with the *pafC* gene. The *pafC* gene was PCR amplified (Table S3) from *P. chrysogenum* Q176 wild-type genomic DNA with the primers *pafC*_fwd_XmaI and *pafC*_rev_BglII (Table S4), containing the restriction sites BglII at the 5' and XmaI at the 3' end, which were attached to the *pafC* gene fragment (Figure S2A). The *pafC* sequence was ligated into the BglII/XmaI digested pSK275_*pafB* vector using the NEBuilder high assembly kit (New England Biolabs, Ipswich, MA, USA), resulting in the plasmid pSK275_*pafC* where *pafC* was put under the regulation of the strong *paf* gene promoter (Figure S2B). The plasmid pSK275_*pafC* was propagated in *E. coli* DH5α, isolated with Monarch Plasmid Miniprep Kit (New England Biolabs, Ipswich, MA, USA) and the nucleotide sequence of the *pafC* expression cassette was verified by Sanger sequencing (Eurofins Genomics,

Ebersberg, Germany). Large plasmid quantities for fungal transformation were purified with PureYield™ Plasmid Midiprep Kit (Promega, Madison, WO, USA).

Transformation pSK275_*pafC* into *P. chrysogenum*

To avoid co-expression of PAF, the NotI-linearized plasmid pSK275_*pafC* was transformed into the *P. chrysogenum* Δ*paf* mutant strain [4] according to the protocol of Cantoral *et al.* (1987) [5] and Kolar *et al.* (1988) [6] and transformants were selected for resistance against 1 µg mL^{-1} pyrithiamine hydrobromide (Sigma-Aldrich, St. Louis, MO, USA) on 1×*Pc*MM agar. The genomic DNA of conidia of growing colonies were then checked for the presence of the fungal resistance marker pyrithiamine (*ptrA*) with PCR (Supplementary Material, Table S3) using the primers *ptrA*_fwd and *ptrA*_rev (Supplementary Material, Table S4). Several positive transformants were selected, grown in 200 mL 1×*Pc*MM and the cell free supernatant was tested for PAFC expression with 18% (w/v) SDS-polyacrylamide gel electrophoresis and silver staining.

Fluorescence microscopy

For localization studies with PAFC-Bd, 1×10^5*Candida* cells were exposed to 1×IC_{90} (2.5 µM) PAFC-Bd for 8 h at 30°C with shaking at 160 rpm and then stained with the nuclei-specific dye Hoechst 33342 (Sigma-Aldrich, St. Louis, MA, USA) in PBS (20 µg mL^{-1} final concentration) for 10 min at room temperature with shaking in the dark.

For the detection of iROS induction by PAFC, 1×10^5 *Candida* cells were incubated with 1×IC_{90} (2.5 µM) PAFC for 8 h at 30°C and shaking at 160 rpm. Untreated cells and cells treated with nystatin (10 µg mL^{-1}) (Sigma-Aldrich, St. Louis, MO, USA) were taken as negative and positive controls, respectively. The cells were then stained with the iROS-specific fluorescent dye H_2DCFDA (Sigma-Aldrich, St. Louis, MO, USA) in PBS (final concentration of 5 µg mL^{-1}), for 30 min at 30°C with shaking at 160 rpm.

Before microscopic analysis all samples were washed twice in PBS and mounted on glass slides for microscopic analysis with a Zeiss Axioplan fluorescence microscope, equipped with an AxioCam MRc camera using excitation/emission filters 265/420 nm for blue fluorescence and 500/535 nm for green fluorescence (Carl Zeiss GmbH, Oberkochen, Germany). Image editing was done with Axiovision (Carl Zeiss GmbH, Oberkochen, Germany), Fiji [7], GNU Image Manipulation Program (GIMP, version 2.8.10) and Microsoft Power Point (Microsoft Corp.).

Supplementary Tables

Table S1. Fungal and bacterial strains used in this study.

Organism	Specification	Source
Candida albicans^{fluS}	fluconazole-sensitive	CBS 5982
Candida albicans^{fluR}	22700, fluconazole-resistant	[8]
Candida glabrata		CBS 138
Candida guilliermondii		CBS 566
Candida krusei		CBS 573
Candida parapsilosis		CBS 604
Escherichia coli DH5α	F^{-} *endA1 glnV44 thi-1 recA1 relA1 gyrA96 deoR nupG purB20* φ80d*lacZ*ΔM15 Δ(*lacZYA-argF*)U169, hsdR17($r_K^- m_K^+$), λ^-	New England Biolabs, Ipswich, MA, USA
Penicillium chrysogenum Q176	wild-type	ATCC 10002
Penicillium chrysogenum Δ*paf*	Δ*paf::nat1*	[9]
Penicillium chrysogenum^{OEpafC}	Δ*paf::nat1, pafC, ptrA*$^{+}$	This study

Table S2. Composition of media and solutions used in this study.

Medium	Abbreviation	Composition/Company[1]
Penicillium chrysogenum minimal medium	1×*Pc*MM	0.3% $NaNO_3$, 0.05% $MgSO_4 \times 7\ H_2O$, 0.05% KCl, 0.005% $FeSO_4 \times 7\ H_2O$, 2% D(+)-sucrose, 2.5% 1 M KPO_4-buffer (pH 5.8), 0.1% trace elements solution *Pc*MM
Penicillium chrysogenum minimal medium double concentrated	2×*Pc*MM	0.6% $NaNO_3$, 0.1% $MgSO_4 \times 7\ H_2O$, 0.1% KCl, 0.01% $FeSO_4 \times 7\ H_2O$, 4% D(+)-sucrose, 2.5% 1 M KPO_4-buffer (pH 5.8), 0.1% trace elements solution *Pc*MM
Trace elements solution *Pc*MM	-	0.1% $FeSO_4 \times 7\ H_2O$, 0.9% $ZnSO_4 \times 7\ H_2O$, 0.04% $CuSO_4 \times 5\ H_2O$, 0.01% $MnSO_4 \times H_2O$, 0.01% H_3BO_3, 0.01% $Na_2MoO_4 \times 2\ H_2O$
Potato dextrose broth	PDB	Sigma-Aldrich, St Louis, MO, USA

[1] Percent values are given as weight per volume (w/v) for solids and volume per volume (v/v) for solutions.

Table S3. PCR conditions applied in this study[§].

Step	Temperature	Time
Initial denaturation	94°C	30 seconds
33 cycles	98°C	10 seconds
	69°C*/70°C[#]	20 seconds[*]/ 30 seconds[#]
	72°C	20 seconds[*] / 1 min 30 seconds[#]
Elongation	72°C	1 minutes[*] / 2 minutes[#]

[§]PCR reaction mix was prepared according to the Q5® High-Fidelity Polymerase reaction protocol (New England Biolabs, Ipswich, MA, USA); [*]PCR conditions for the *pafC* amplicon; [#]PCR conditions for the *ptrA* amplicon.

Table S4. Oligonucleotides used in this study.

Primer	Sequence 5'-3'	Amplicon
*pafC*_fwd_XmaI	ACC ATC CCG GGC TAG CAT CTG GCT CCC CC	
*pafC*_rev_BglII	CAT AAA GAT CTA TGA AGG TTA CTG CTC TCC TCT TCA CCC	*pafC* gene
*ptrA*_fwd	GCA CTG AAC CCA TTC GGG TAG TGA G	*ptrA* resistance
*ptrA*_rev	CGG TGT TCG TTC CCA GTC ATC G	cassette

Supplementary Figures

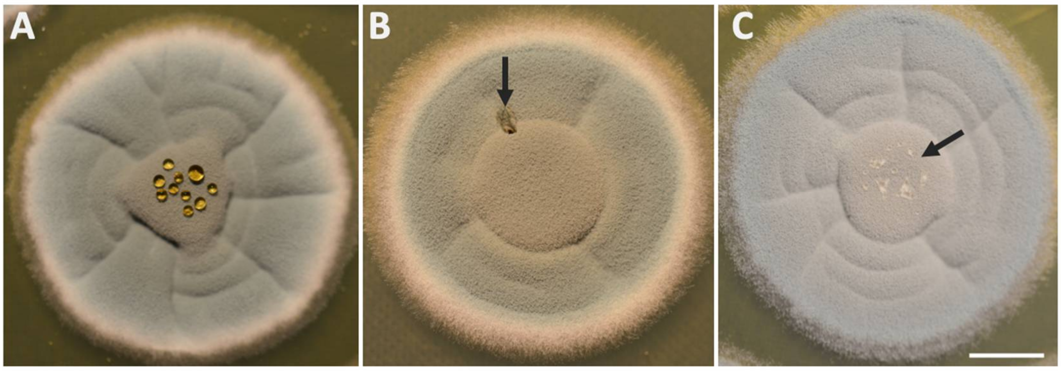

Figure S1. Exudate formation of *P. chrysogenum* surface colonies on 2×*Pc*MM agar. (A) Droplets on top of the colony after cultivation for 120 h at 25°C. **(B)** 120 h-old surface colony after harvesting the exudate accumulated under the colony. The arrow indicates the puncture for the exudate harvest. **(C)** Footprints of droplets on top of the colony after incubation for 144 h at 25°C are indicated by an arrow. Scale bar, 5 mm.

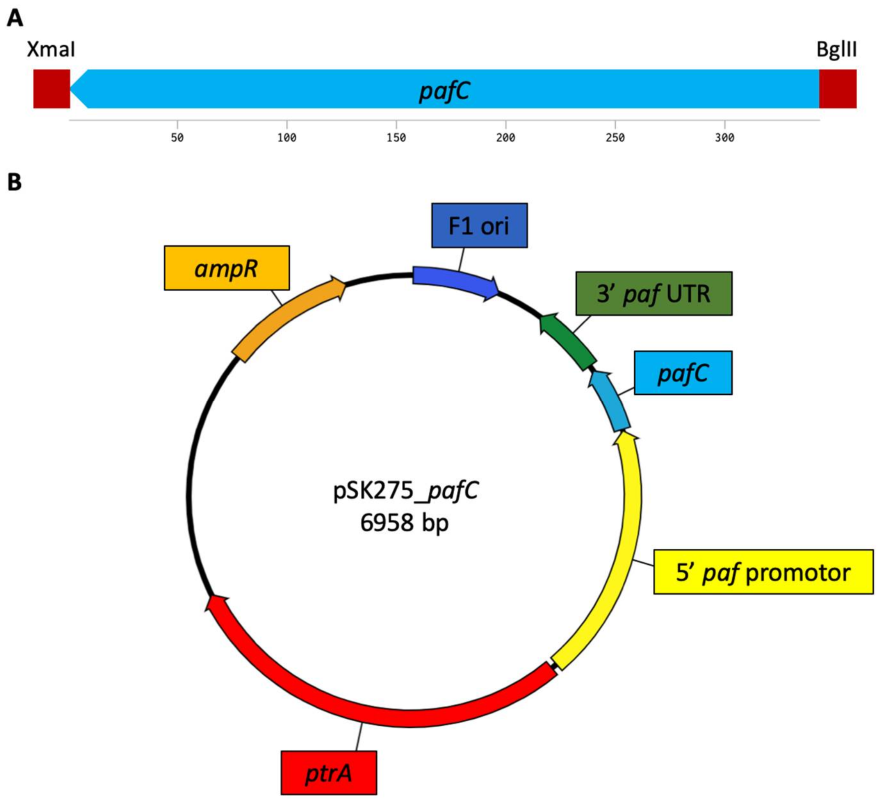

Figure S2. Cloning of the expression vector pSK275_*pafC*. (A) Schematic representation of the *pafC* PCR fragment (turquoise) with attached restriction sites BglII and XmaI (red). **(B)** Map of the expression plasmid pSK275_*pafC* used for transformation of *P. chrysogenum* Δ*paf*. The *pafC* gene expression cassette consists of the *paf* promotor (5' *paf* promotor, yellow), the *pafC* gene fragment (*pafC*, turquoise) and the 3' terminator of *paf* (3' *paf* UTR, green). The plasmid further contains the origin of replication (F1 ori, blue), the ampicillin resistance gene (*ampR*, orange) and the pyrithiamine resistance gene (*ptrA*, red).

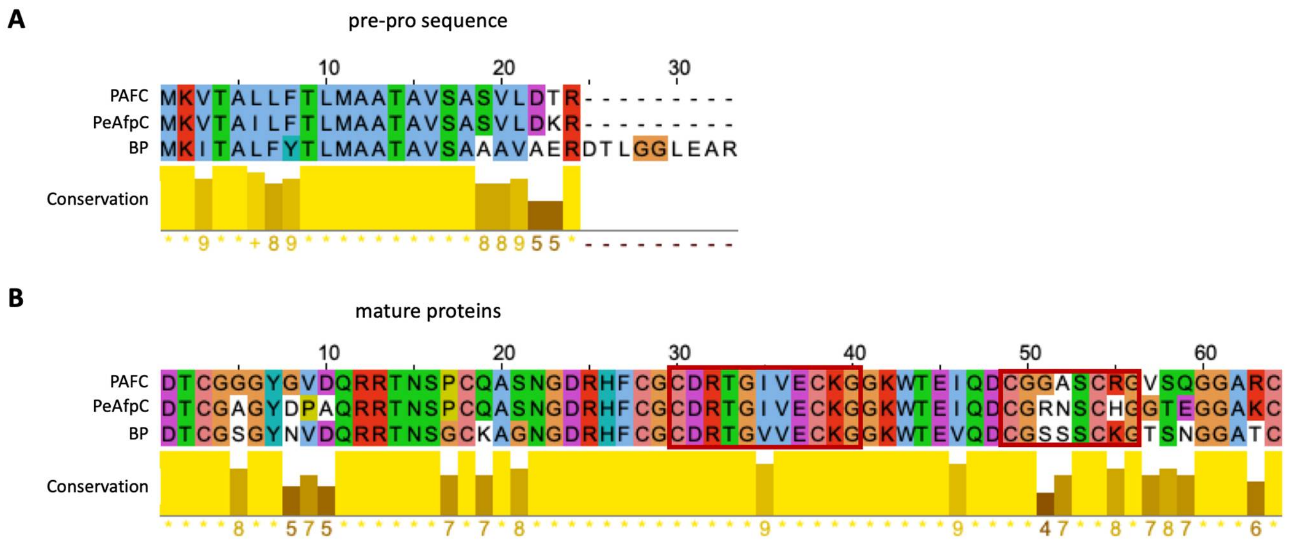

Figure S3. Amino acid alignment of PAFC and orthologous AMPs. (A) pre-pro sequence and **(B)** mature protein sequence of the *P. chrysogenum* PAFC [2], the *P. expansum* PeAfpC [3] and the *P. brevicompactum* BP [10] including the respective conservation between the sequences based on the ClustalW2 Multiple Sequence Alignment tool [11]. Conserved regions are marked with an asterisk, scale is 1-10. The γ-core motifs are framed in red. Color coding of amino acids according to ClustalX.

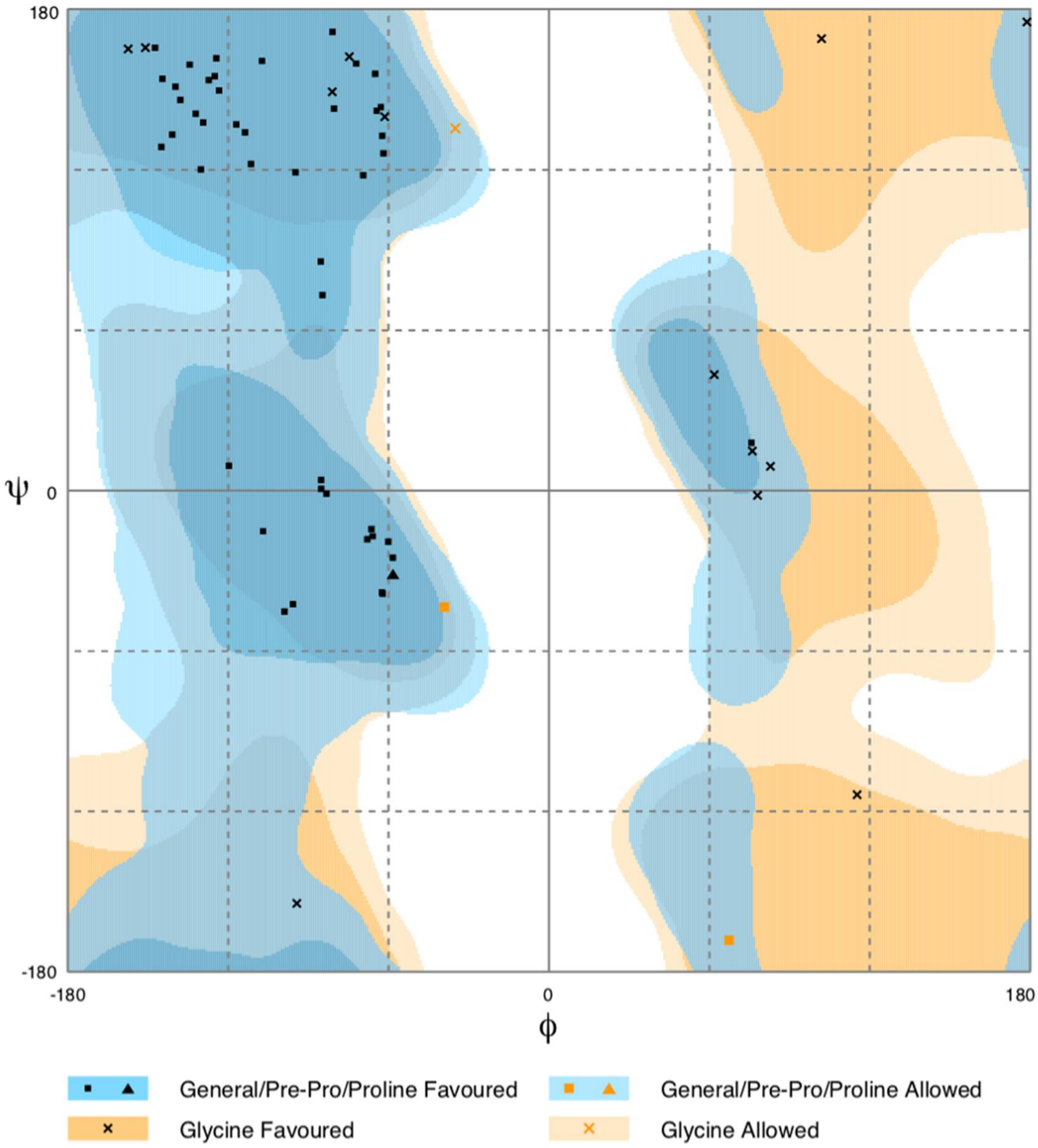

Figure S4. Ramachandran plot of the PAFC model. Energetically allowed regions for backbone dihedral angles ψ against φ of the amino acid residues in PAFC according to the RAMPAGE server tool [12]. General amino acids are depicted with a square, proline residues with a triangle and glycine residues with a cross. Favored regions are indicated in dark orange for glycine, other amino acids in dark blue, allowed regions in light orange for glycine and light blue for all other amino acids. Amino acids in energetically favored regions are shown in black (95.2%), and in allowed regions in orange (4.8%). No amino acids are in the outlier region (0%).

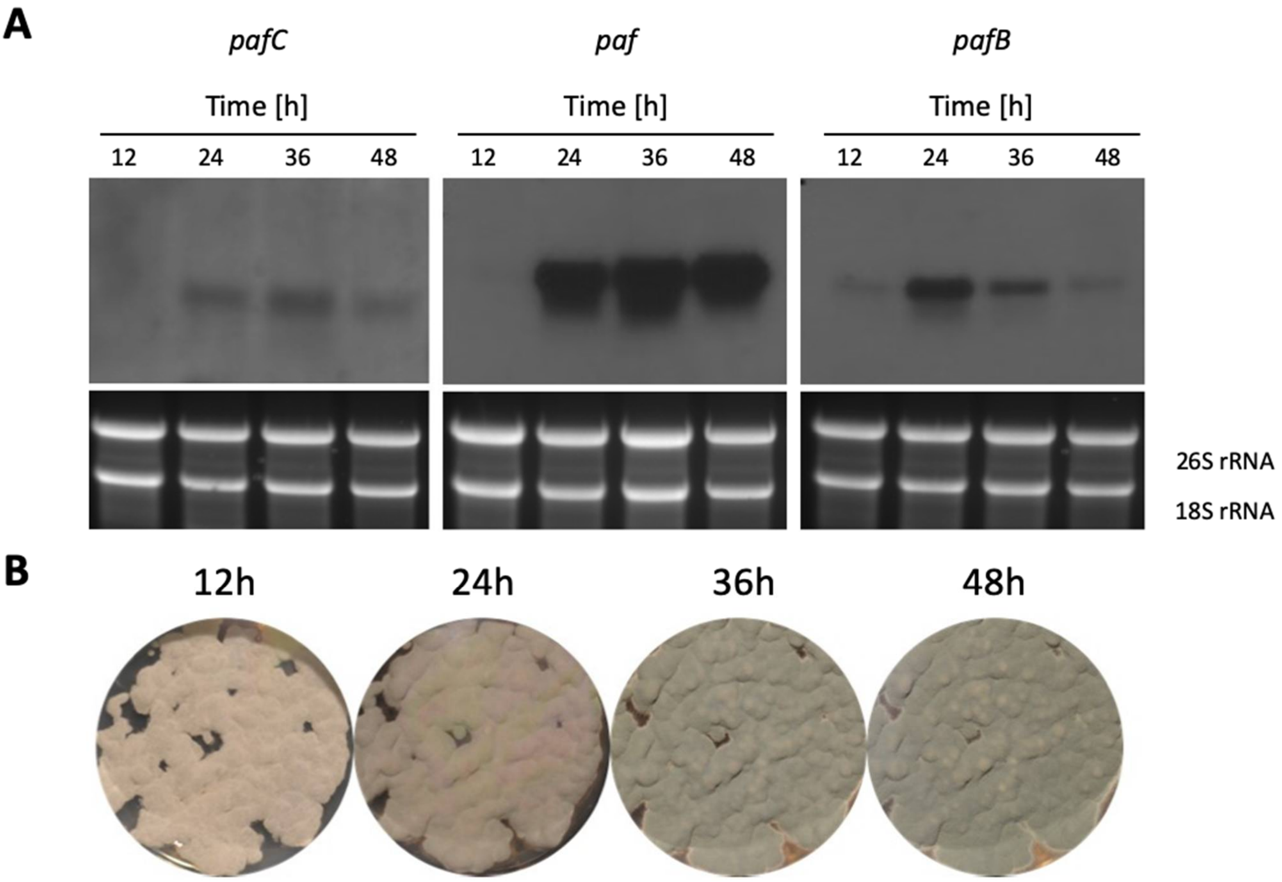

Figure S5. **Expression of *pafC, paf* and *pafB* in synchronized surface cultures of *P. chrysogenum* over a time course of 12-48 h of incubation. (A)** Ten µg of total RNA was loaded on a 1.2 % (w/v) formaldehyde agarose gel, blotted and hybridized with the respective gene specific digoxigenin-labelled probes [2] (upper panel). Ethidium bromide stained 26S and 18S rRNA provide loading controls (lower panels). **(B)** Images of synchronized surface cultures of *P. chrysogenum* grown on 1×*Pc*MM over a time course of 12-48 h. The onset of sporulation was at 24 h of incubation.

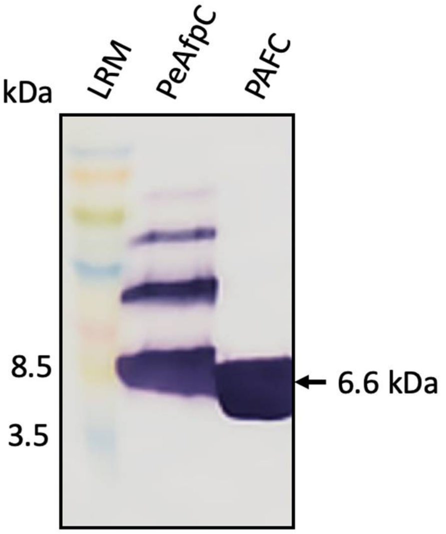

Figure S6. Western blot analysis to prove the binding of the PeAfpC antibody to PAFC. Per lane, 5 µg of PeAfpC and PAFC were loaded on a 18% (w/v) SDS-polyacrylamide gel, size fractionated and transferred onto a nitrocellulose membrane. Polyclonal rabbit PeAfpC antibody (1:2500) and enzyme-linked secondary anti-rabbit IgG (1:10000, Sigma-Aldrich, St. Louis, MA, USA) were used for the detection of PAFC. Visualization of antigen-antibody complexes was performed with BCIP/NBT system (Promega, Madison, WO, USA). Low range rainbow marker (LRM) (GE Healthcare Life Sciences, Little Chalfont, UK) was used as size marker. The molecular weight (MW) marker bands of 3.5 kDa and 8.5 kDa are indicated and the MW of PAFC (6.6 kDa) is marked with an arrow.

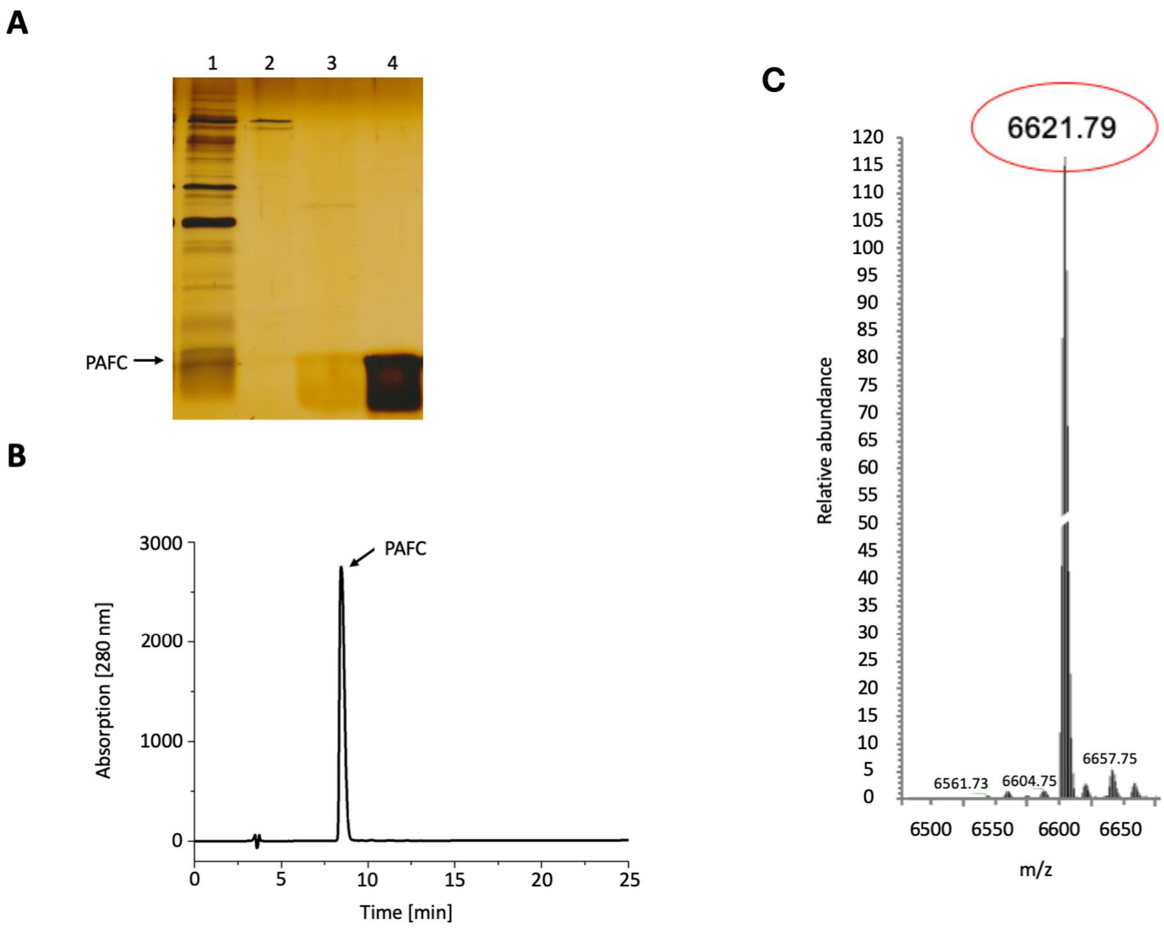

Figure S7. Purification of recombinant PAFC. (A) 18% (w/v) silver stained SDS-polyacrylamide gel showing samples collected during the PAFC purification process. Lanes: (1) Crude 96-hours-old cell-free supernatant; (2) column wash (25 mM NaCl); (3) eluted fraction 3 (100 mM NaCl); (4) eluted fraction 6 (100 mM NaCl) containing PAFC. PAFC is indicated by an arrow. **(B)** Reversed-phase high-performance liquid chromatography analysis of PAFC. The PAFC specific peak is indicated by an arrow. **(C)** The protein identity was confirmed with electro-spray mass spectrometry. The molecular mass in Da corresponding to PAFC (oxidized form) is circled in red.

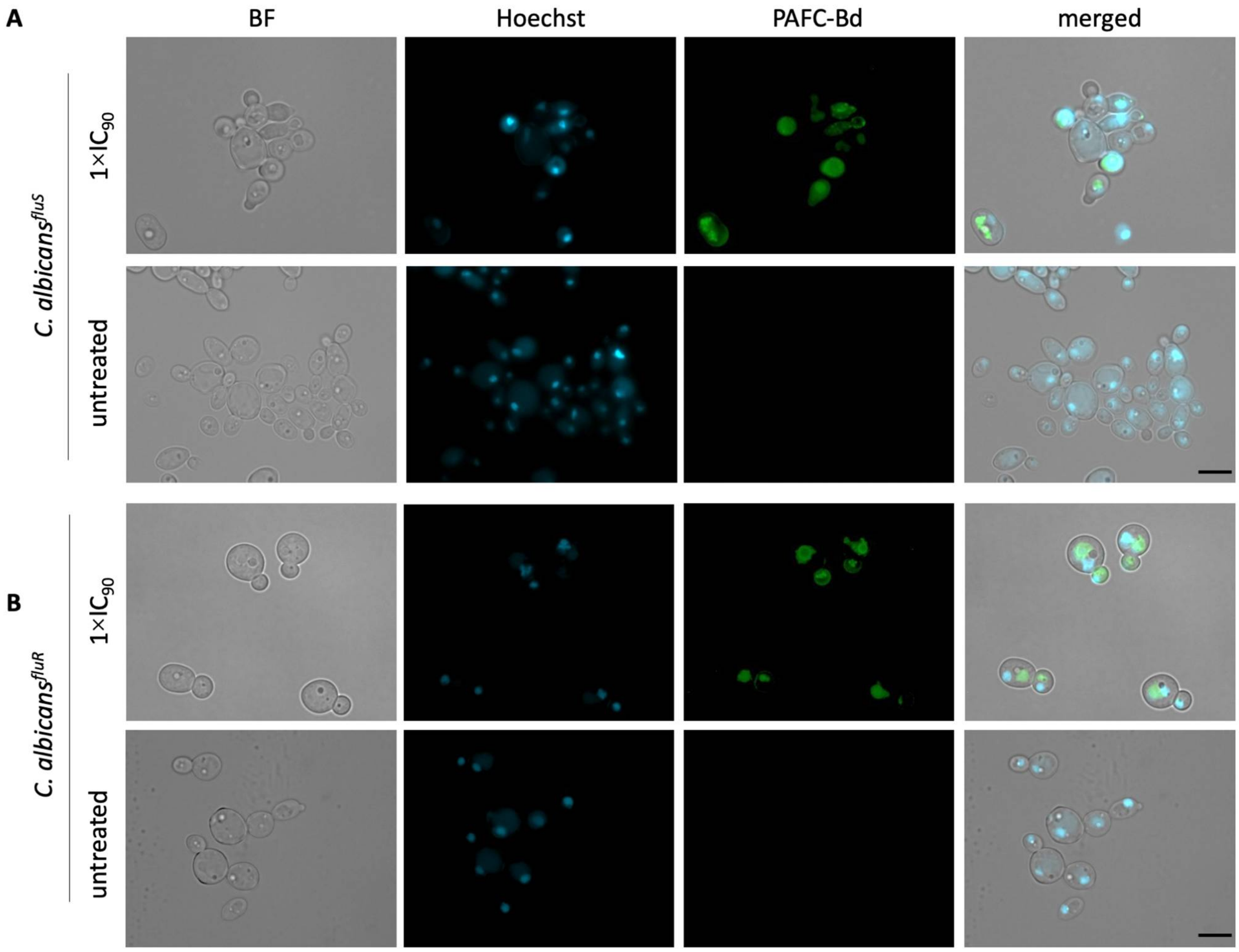

Figure S8. Fluorescence microscopy for the localization of nuclei and PAFC-Bd. (A) *C. albicans*^{fluS} and **(B)** *C. albicans*^{fluR} were exposed to 1×IC_{90} PAFC-Bd (2.5 µM) for 8 h and then nuclei were stained with the nuclei-specific dye Hoechst 33342 (20 µg mL^{-1}) for 10 min in the dark before microscopic analysis. Cells without PAFC-Bd treatment (untreated) were used as controls. The merged images show the nuclei-specific and the PAFC-Bd fluorescent signals superimposed in the *Candida* cells visualized with brightfield microscopy. One representative image out of three replicates is shown. BF = brightfield, PAFC-Bd = Bd-labelled PAFC. Scale bar, 5 µm.

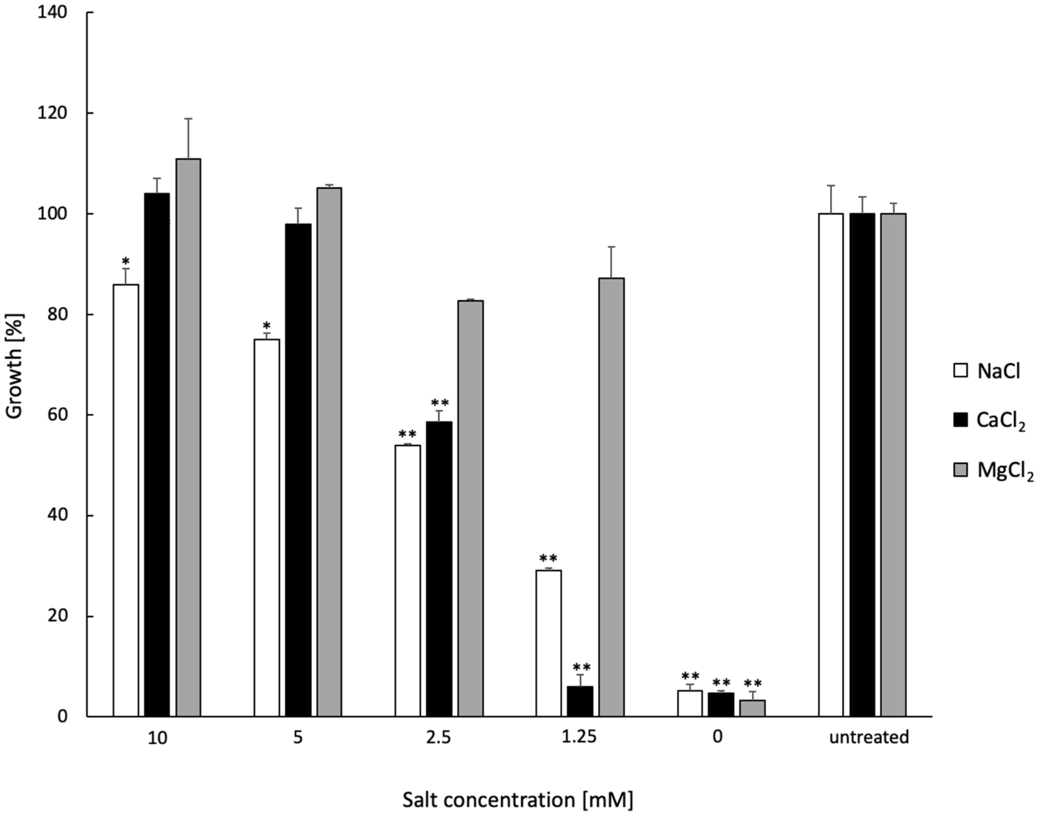

Figure S9. Ion tolerance of PAFC. The activity of 1×IC_{90} (2.5 µM) PAFC was tested on *C. albicans*^{fluS} in the presence of increasing concentrations (0 -10 mM) of NaCl, $CaCl_2$ and $MgCl_2$ in a microdilution broth assay. *Candida* cells left untreated were used as growth control representing 100% growth. The mean ± SD (technical triplicate of one representative experiment out of two biological replicates) is shown. A two-sample Student's t-test was applied to calculate the significant difference between the salt-treated samples compared to the untreated controls (no PAFC) ($^{*}p \leq 0.05$ and $^{**}p \leq 0.005$).

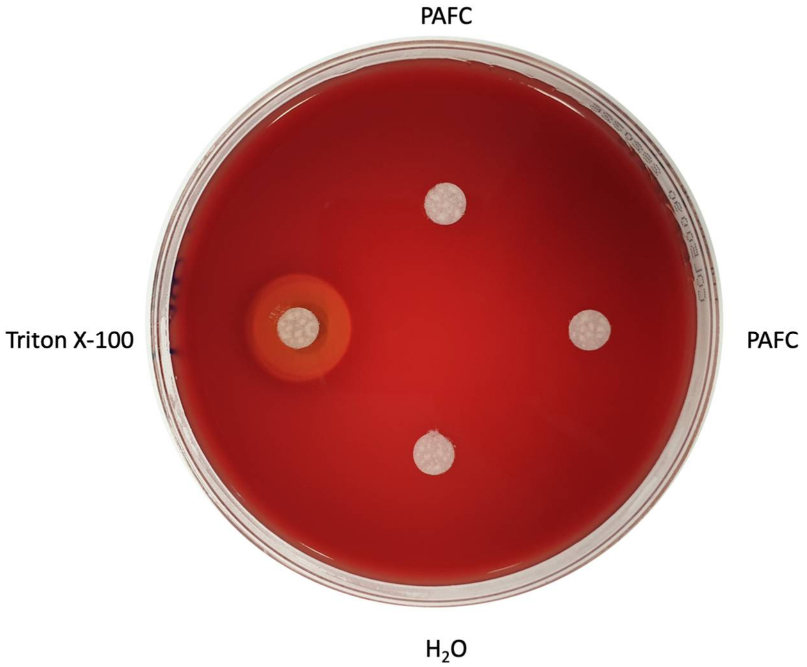

Figure S10. Hemolytic activity of PAFC tested with agar diffusion assay. PAFC was added in 10 µL aliquots containing 13 µg protein on sterile filter discs on Columbia blood agar plates. Water was used as a negative control and 20% (v/v) Triton X-100 as a positive control for induction of hemolysis. The plates were incubated for 24 h at 37°C. One representative experiment out of two.

Supplementary References

1. Oberparleiter, C.; Kaiserer, L.; Haas, H.; Ladurner, P.; Andratsch, M.; Marx, F. Active internalization of the *Penicillium chrysogenum* antifungal protein PAF in sensitive *Aspergilli*. *Antimicrob Agents and Chemother* **2003**, *47*, 3598-3601, doi:10.1128/aac.47.11.3598-3601.2003.
2. Huber, A.; Hajdu, D.; Bratschun-Khan, D.; Gáspári, Z.; Varbanov, M.; Philippot, S.; Fizil, A.; Czajlik, A.; Kele, Z.; Sonderegger, C.; et al. New antimicrobial potential and structural properties of PAFB: a cationic, cysteine-rich protein from *Penicillium chrysogenum* Q176. *Sci Rep* **2018**, *8*, 1751, doi:10.1038/s41598-018-20002-2.
3. Garrigues, S.; Gandía, M.; Castillo, L.; Coca, M.; Marx, F.; Manzanares, P.; Marcos, J.F.; Manzanares, P. Three antifungal proteins from *Penicillium expansum* : Different patterns of production and antifungal activity. *Front Microbiol* **2018**, *9*, 2370, doi: 10.3389/fmicb.2018.02370.
4. Sonderegger, C.; Galgóczy, L.; Garrigues, S.; Fizil, Á.; Borics, A.; Manzanares, P.; Hegedüs, N.; Huber, A.; Marcos, J.F.; Batta, G.; et al. A *Penicillium chrysogenum*-based expression system for the production of small, cysteine-rich antifungal proteins for structural and functional analyses. *Microb Cell Fact* **2016**, *15*, 192, doi: 10.1186/s12934-016-0586-4.
5. Cantoral, J.M.; Díez, B.; Barredo, J.; Alvarez, E.; Martín, J.F. High–frequency transformation of *Penicillium chrysogenum*. *Nat Biotechnol* **1987**, *5*, 494-497, doi.org/10.1038/nbt0587-494.
6. Kolar, M.; Punt, P.J.; van den Hondel, C.A.; Schwab, H. Transformation of *Penicillium chrysogenum* using dominant selection markers and expression of an *Escherichia coli lacZ* fusion gene. *Gene* **1988**, *62*, 127-134, doi: 10.1016/0378-1119(88)90586-0.
7. Schindelin, J.; Arganda-Carreras, I.; Frise, E.; Kaynig, V.; Longair, M.; Pietzsch, T.; Preibisch, S.; Rueden, C.; Saalfeld, S.; Schmid, B.; et al. Fiji: an open-source platform for biological-image analysis. *Nat Methods* **2012**, *9*, 676-682, doi: 10.1038/nmeth.2019.
8. Kovács, R.; Holzknecht, J.; Hargitai, Z.; Papp, C.; Farkas, A.; Borics, A.; Tóth, L.; Váradi, G.; Tóth, G.; Kovács, I; et al. *In vivo* applicability of *Neosartorya fischeri* antifungal protein 2 (NFAP2) in treatment of vulvovaginal candidiasis. *Antimicrob Agents Chemother* **2019**, *63*, e01777-18, doi:10.1128/AAC.01777-18.
9. Hegedüs, N.; Sigl, C.; Zadra, I.; Pócsi, I., Marx, F. The *paf* gene product modulates asexual development in *Penicillium chrysogenum*. *J Basic Microbiol* **2011**, *51*, 253-262, doi:10.1002/jobm.201000321.
10. Olsen, J.G.; Flensburg, C.; Olsen, O.; Bricogne, G.; Henriksen, A. Solving the structure of the bubble protein using the anomalous sulfur signal from single-crystal in-house Cu Kalpha diffraction data only. *Acta Crystallogr D Biol Crystallogr* **2004**, *60*, 250-255, doi: 10.1107/S0907444903025927.
11. Sievers, F.; Wilm, A.; Dineen, D.; Gibson, T.J.; Karplus, K.; Li, W.; Lopez, R.; McWilliam, H.; Remmert, M.; Söding, J.; et al. Fast, scalable generation of high-quality protein multiple sequence alignments using Clustal Omega. *Mol Syst Biol* **2011**, *7*, 539, doi: 10.1038/msb.2011.75.
12. Lovell, S.C.; Davis, I.W.; Arendall, W.B., 3rd; de Bakker, P.I.; Word, J.M.; Prisant, M.G.; Richardson, J.S.; Richardson, D.C. Structure validation by Calpha geometry: phi, psi and Cbeta deviation. *Proteins* **2003**, *50*, 437-450, doi:10.1002/prot.10286.
