# Supplementary figures and images for: The Penicillium chrysogenum Q176 Antimicrobial Protein PAFC Effectively Inhibits the Growth of the Opportunistic Human Pathogen Candida albicans
Source: J Fungi (Basel). 2020 Aug 19;6(3):141. doi: 10.3390/jof6030141 (PMC7557831; doi:10.3390/jof6030141)

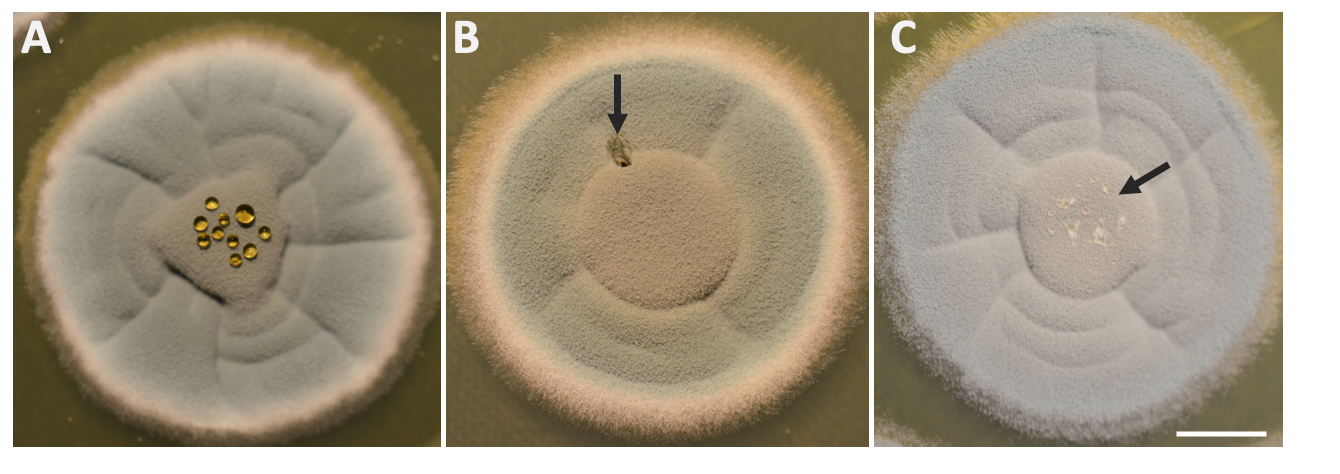

Supplement: Supplementary file 1 [file jof-06-00141-s001.zip › SupplementaryFigures_JoF/Figure S1.tiff]

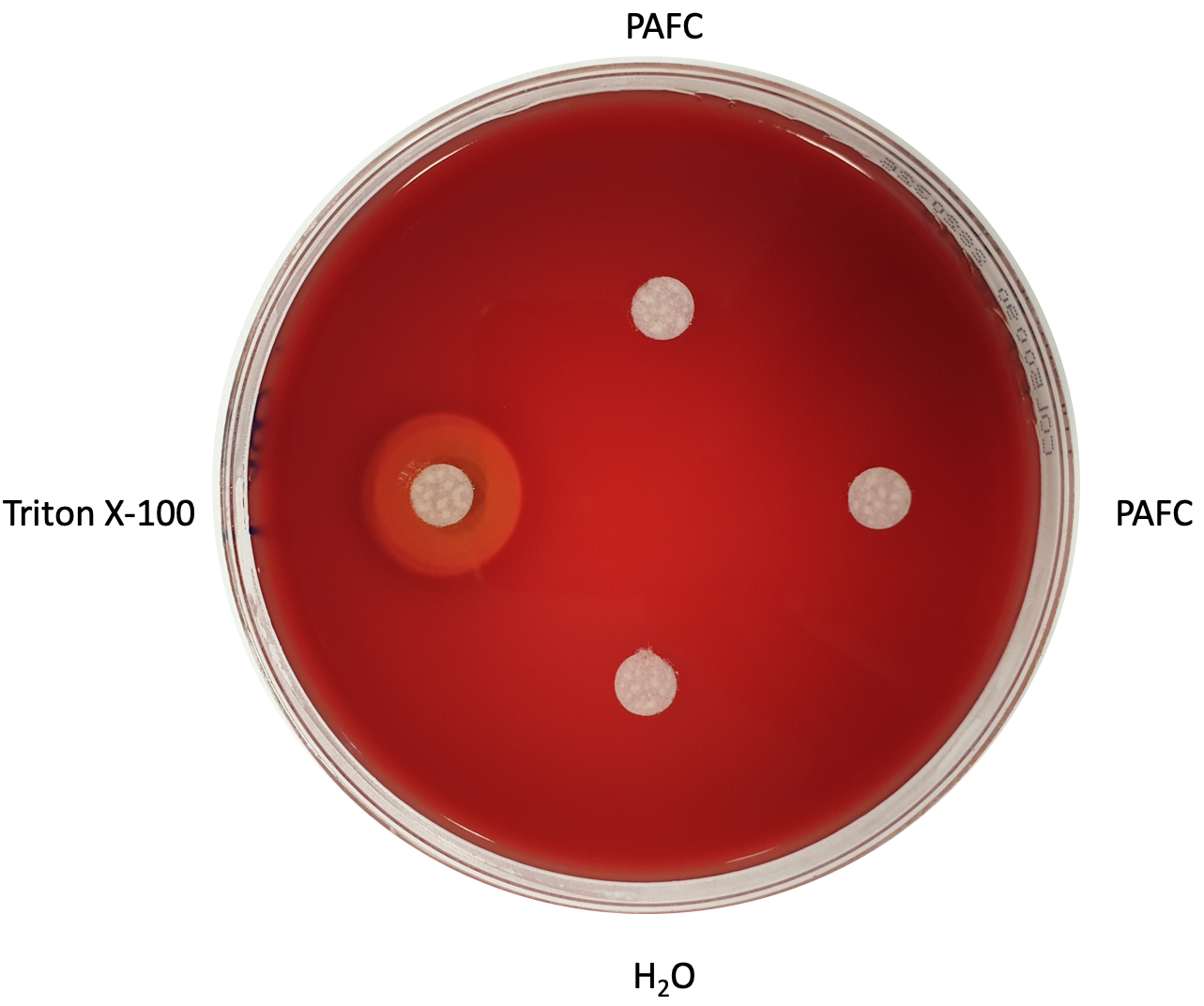

Supplement: Supplementary file 1 [file jof-06-00141-s001.zip › SupplementaryFigures_JoF/Figure S10.tiff]

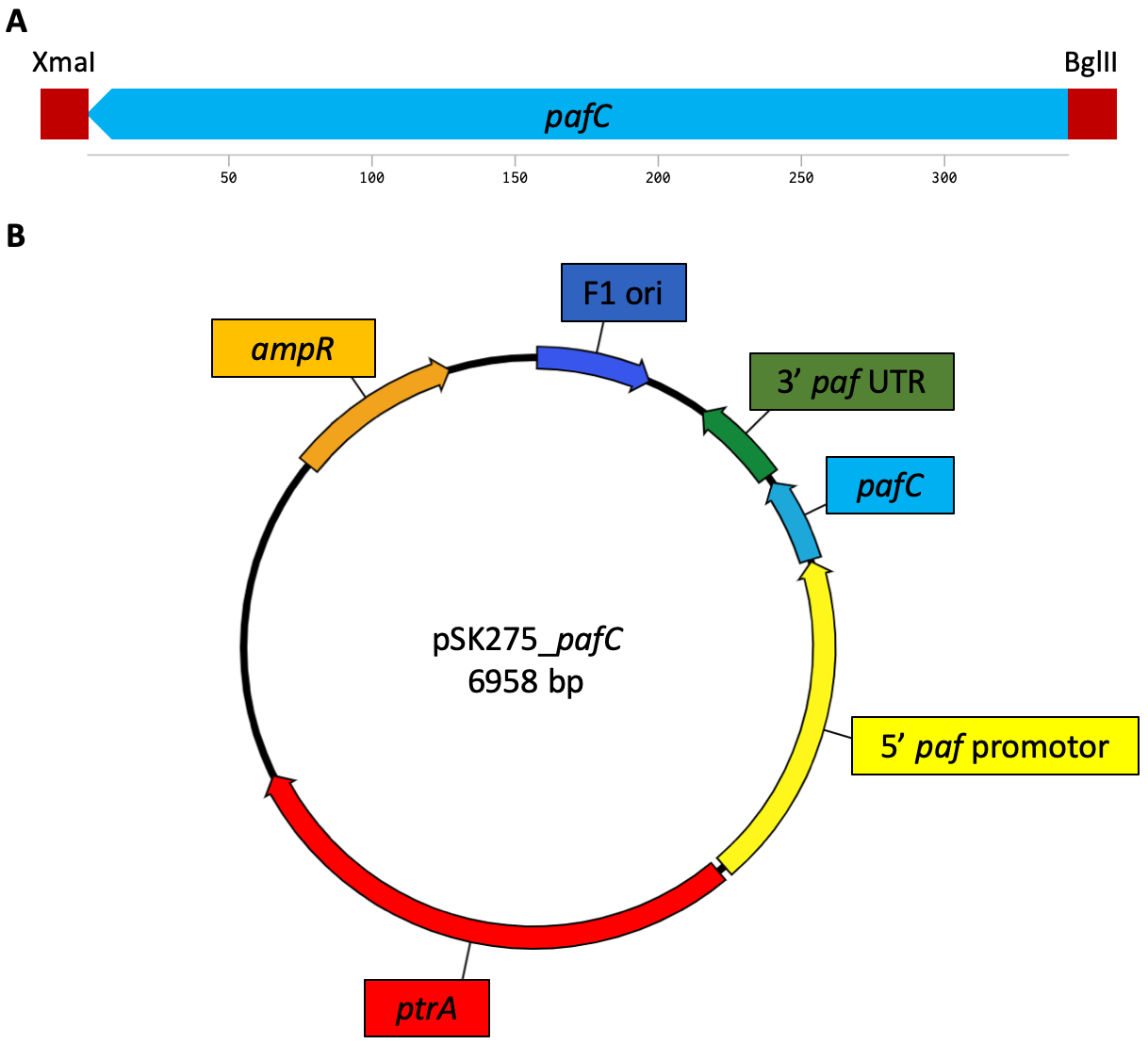

Supplement: Supplementary file 1 [file jof-06-00141-s001.zip › SupplementaryFigures_JoF/Figure S2.tiff]

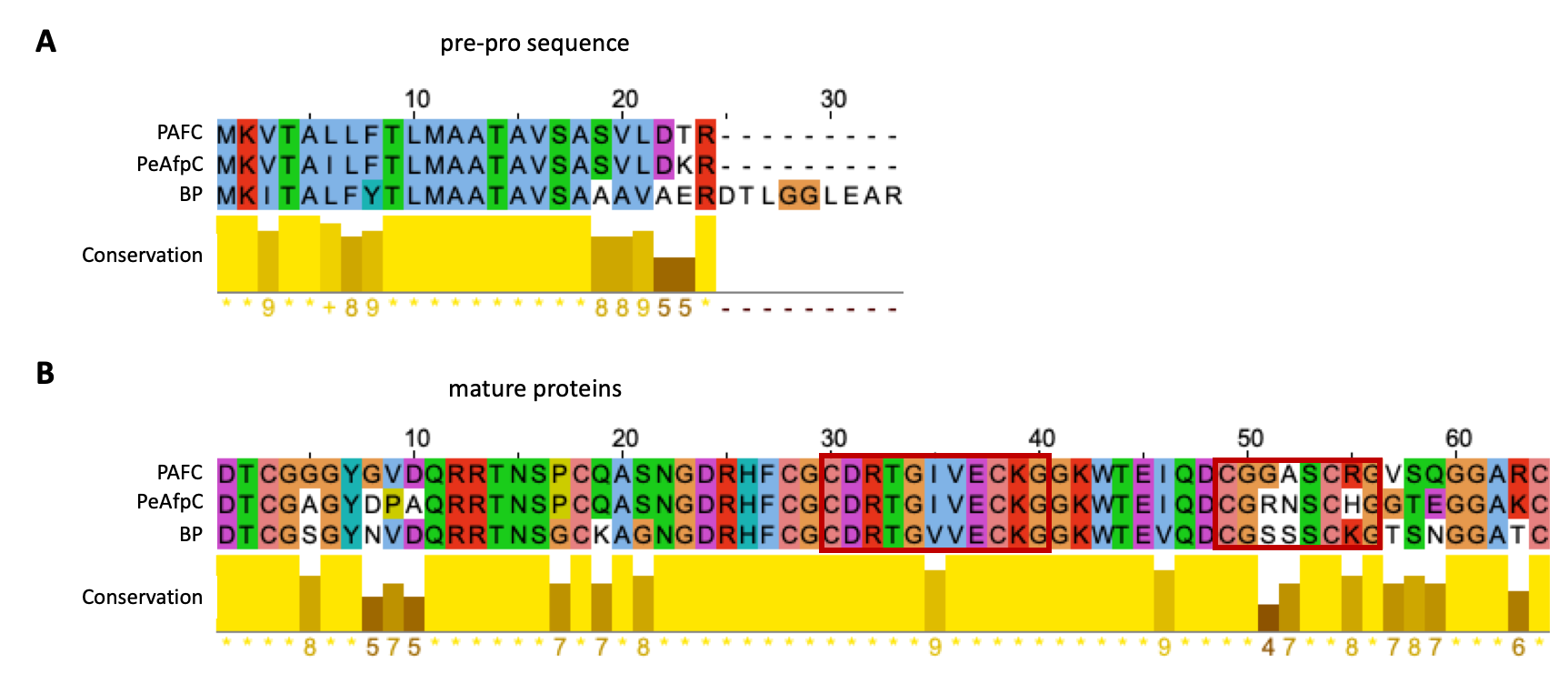

Supplement: Supplementary file 1 [file jof-06-00141-s001.zip › SupplementaryFigures_JoF/Figure S3.tiff]

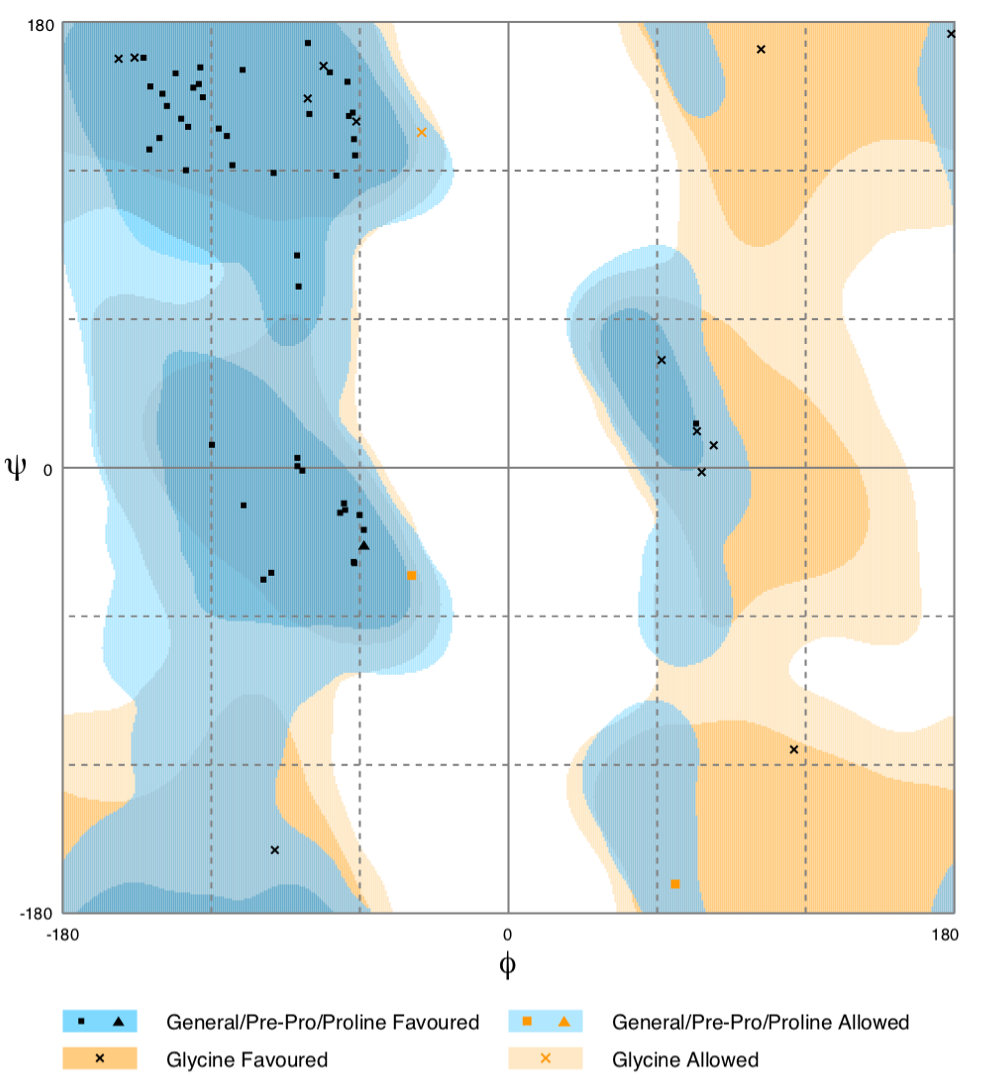

Supplement: Supplementary file 1 [file jof-06-00141-s001.zip › SupplementaryFigures_JoF/Figure S4.tiff]

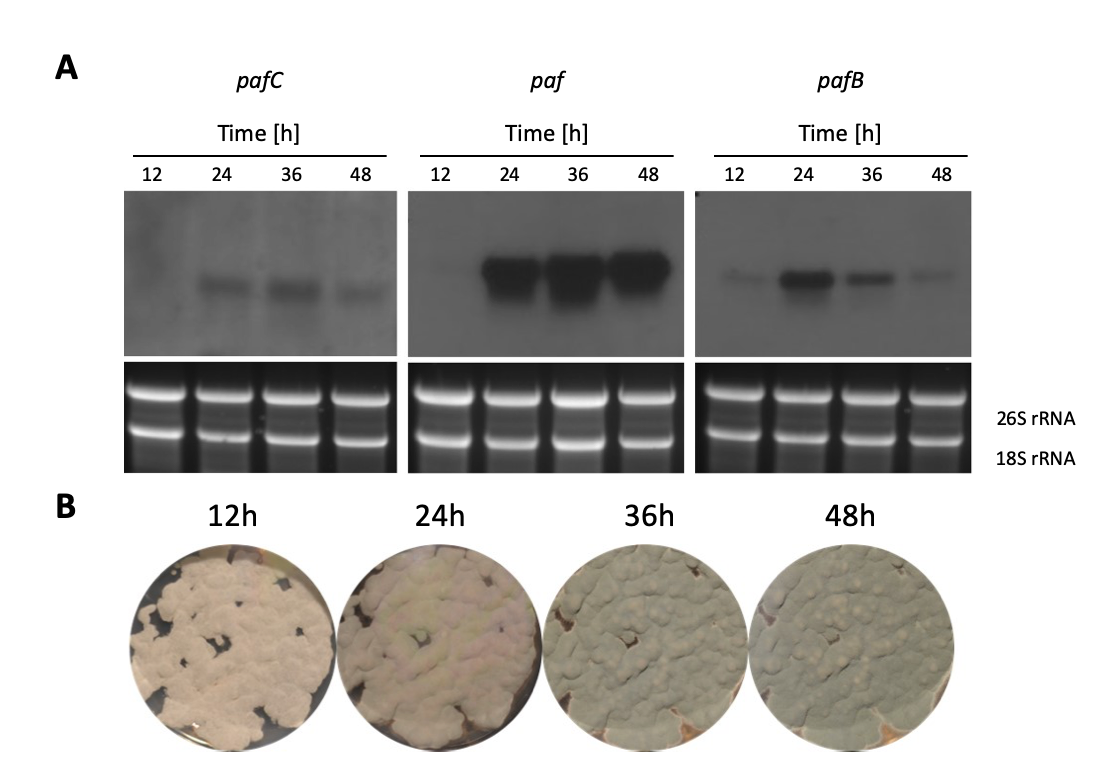

Supplement: Supplementary file 1 [file jof-06-00141-s001.zip › SupplementaryFigures_JoF/Figure S5.tiff]

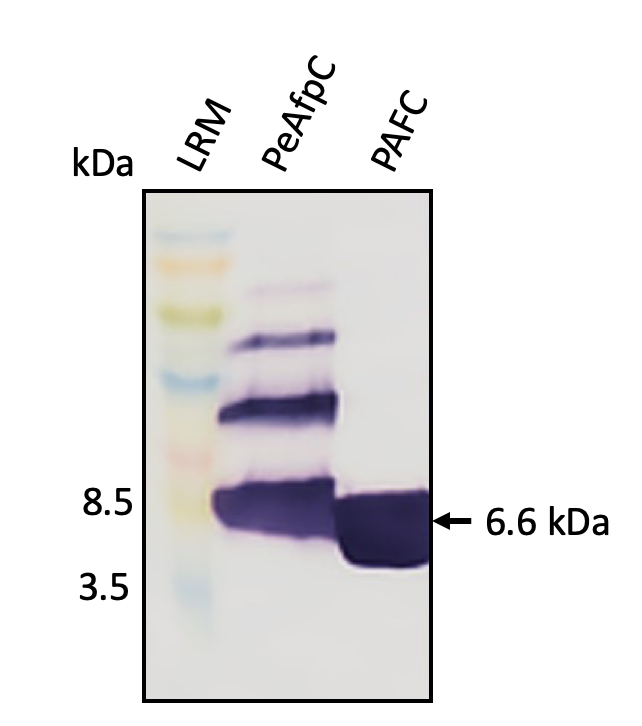

Supplement: Supplementary file 1 [file jof-06-00141-s001.zip › SupplementaryFigures_JoF/Figure S6.tiff]

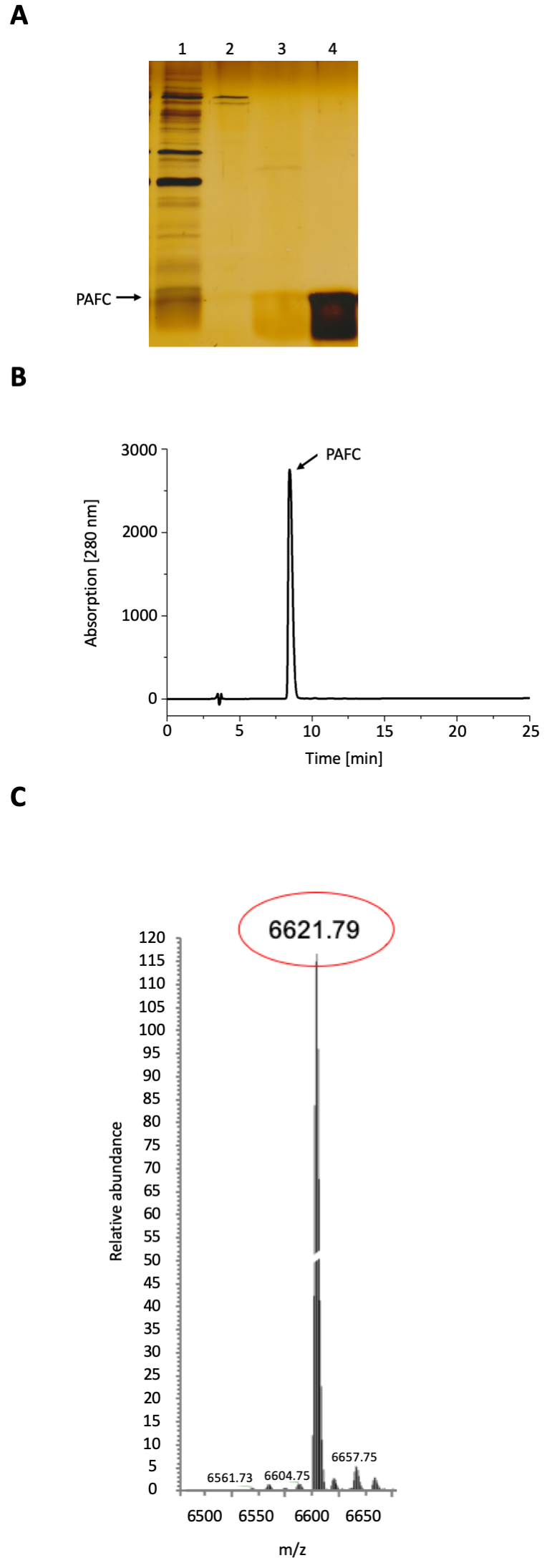

Supplement: Supplementary file 1 [file jof-06-00141-s001.zip › SupplementaryFigures_JoF/Figure S7.tiff]

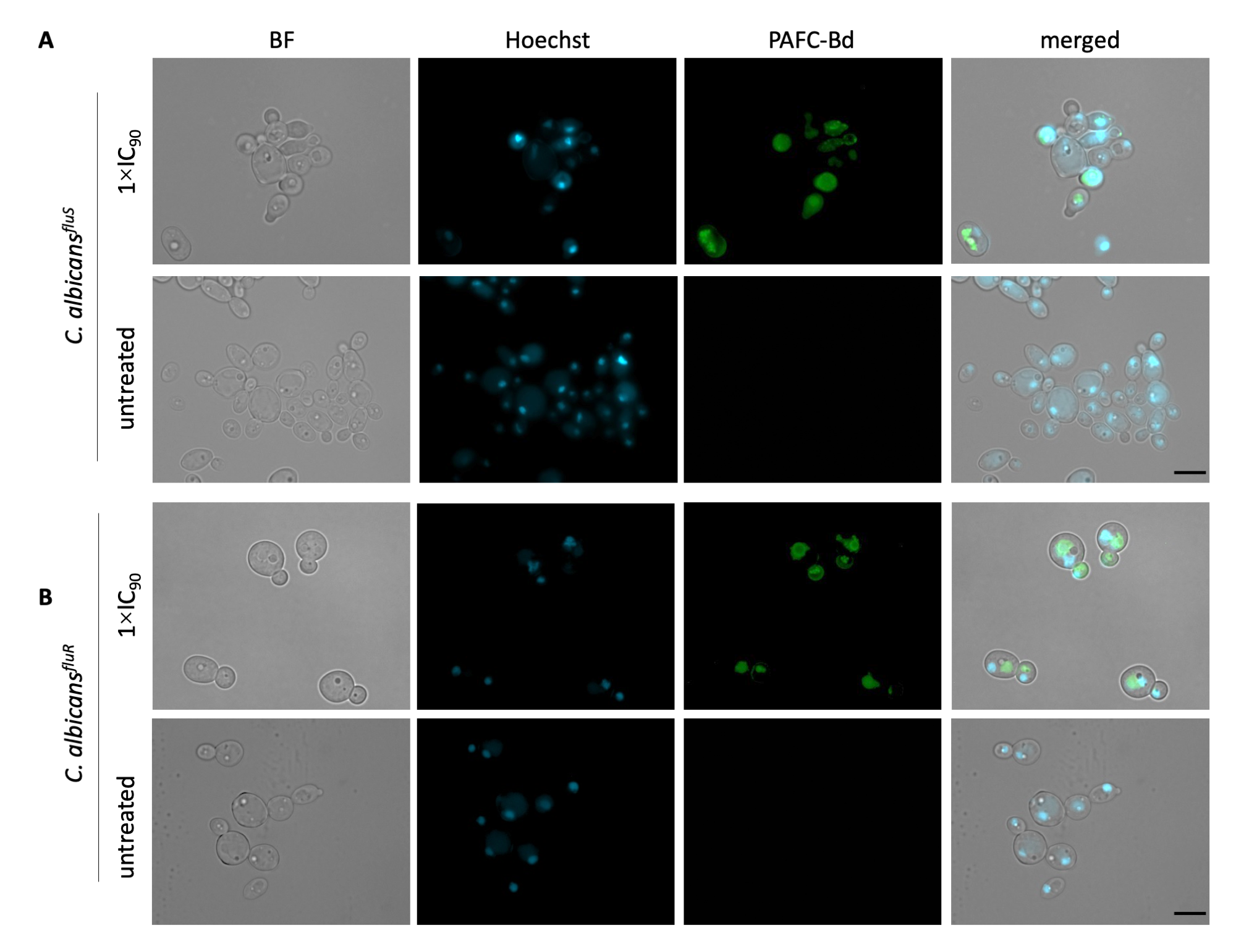

Supplement: Supplementary file 1 [file jof-06-00141-s001.zip › SupplementaryFigures_JoF/Figure S8.tiff]

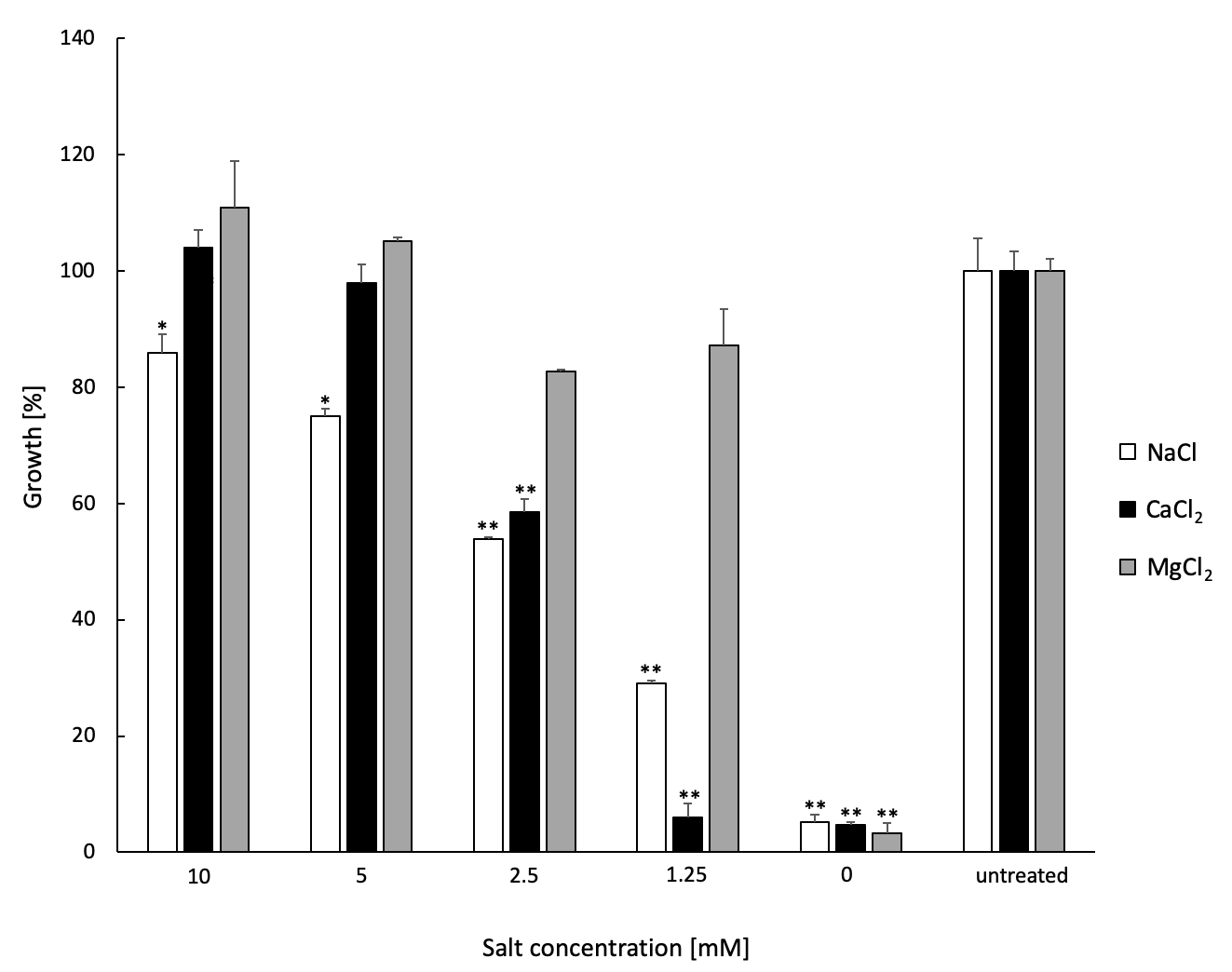

Supplement: Supplementary file 1 [file jof-06-00141-s001.zip › SupplementaryFigures_JoF/Figure S9.tiff]
